# Exploring motivations, barriers and solutions for interdisciplinary practice in work-focused healthcare: a qualitative study among Dutch healthcare professionals

**DOI:** 10.1136/bmjopen-2025-103881

**Published:** 2026-03-19

**Authors:** Nina Zipfel, Ersen Colkesen, Marije E Hagendijk, Marijke Melles, Sylvia J van der Burg-Vermeulen

**Affiliations:** 1 Department of Public and Occupational Health, Amsterdam UMC Location University of Amsterdam, Amsterdam, Netherlands; 2 Societal Participation & Health, Amsterdam Public Health Research Institute, Amsterdam, Netherlands; 3 Department of Cardiology, St Antonius Ziekenhuis, Nieuwegein, Netherlands; 4 Delft University of Technology Faculty of Industrial Design Engineering, Delft, ZH, Netherlands

**Keywords:** Occupational Health Services, QUALITATIVE RESEARCH, Patient-Centered Care

## Abstract

**Abstract:**

**Objectives:**

To explore the motivations, barriers and potential solutions from professionals’ perspectives for achieving effective interdisciplinary practice, focusing on communication and collaboration to support work participation and facilitate patients’ return-to-work (RTW).

**Design:**

Qualitative exploratory interview study using thematic analysis.

**Setting:**

Primary and work-focused healthcare.

**Participants:**

22 healthcare professionals, including occupational physicians (n=5), social insurance physicians (n=5), general practitioners (n=7) and occupational physiotherapists (n=5), were purposively recruited. All participants had at least 1 year of experience and were actively involved in patient care.

**Interventions:**

None.

**Primary outcome measures:**

Identified motivations, experienced barriers and proposed solutions for improving communication and collaboration across disciplines in work-focused healthcare.

**Results:**

Participating healthcare professionals emphasised the importance of early, proactive and person-centred communication across care domains, particularly in complex or stagnating RTW trajectories. Key barriers included a lack of shared knowledge and common goals, limited understanding of each other’s roles, time constraints, fragmented systems and regulatory restrictions. Proposed solutions focused on clearer coordination of care, improved role clarity and development of a secure, cross-domain digital communication platform to streamline information exchange.

**Conclusions:**

Effective work-focused healthcare requires improved interprofessional communication and patient-centred collaboration. This study highlights when collaboration is most valuable, identifies key barriers and outlines feasible practical solutions. Future research should focus on developing and implementing guidelines that clarify communication pathways and coordination mechanisms within interdisciplinary teams.

**STRENGTH AND LIMITATION OF THE STUDY**

- The study used purposive sampling across four key healthcare professional groups to ensure a diverse range of perspectives on work-focused healthcare.
- Thematic analysis was conducted using a multi-step coding and review process involving multiple researchers, enhancing the credibility and consistency of the findings.
- Interviews were conducted by supervised master’s students, and measures were taken (eg, oral debriefings and written summaries) to ensure accurate contextual transfer to the research team.
- The interview guide was used consistently across teams, but inter-interviewer variability may have influenced the depth or style of questioning during data collection.
- Interviews were conducted in both Dutch and English, which may have limited nuanced expression for some participants, although language preferences were communicated in advance.

## Background

 The concept of health underwent a significant transformation in 2009 when Huber *et al* redefined it, shifting from the mere absence of disease to emphasise ‘the ability to adapt and to self-manage in the face of social, physical and emotional challenges’.^1^ This redefinition moved the focus of healthcare beyond diagnosis and cure by putting greater emphasis on personal functioning, participation in the labour market and the dynamic interaction between individuals and their environment.^1^ Here, adaptation refers to a person’s capacity to adjust to physical limitations, emotional stressors or social barriers, and self-management means the ability to take control of one’s own health, including decision-making, self-monitoring and interaction with care providers.^2 3^ This paradigm shift highlights the importance of social participation as a key determinant of health, paving the way for subsequent developments in healthcare policy and practice. The Royal Dutch Medical Association embedded this perspective in its 2017 vision for participation-oriented healthcare, emphasising work participation as a crucial aspect of health and identifying sustainable work capacity as a key goal.^4^ In this context, high-quality and accessible work-focused healthcare is considered essential. In this context, work-focused healthcare is defined as the advice, treatment, guidance and support received by patients with work participation problems, from both occupational health professionals and curative care professionals.^5^ These healthcare services take into account an individual’s ability to engage in work, with the aim of supporting sustainable employability and aligning with broader health and participation objectives.^5^ This broad scope distinguishes it from occupational healthcare, which often is limited to services provided by occupational physicians.^5^ Sustainable employability refers to the synthesis of opportunities, conditions and a supportive work environment that enables individuals to remain engaged in work while preserving their health and welfare throughout their working life.^6^ This concept aligns with the United Nations Sustainable Development goals which aim, among others, to ‘promote sustained, inclusive and sustainable economic growth, full and productive employment and decent work for all’.^7^ The 2030 Agenda defines decent work as including employment creation, social protection, rights at work and social dialogue^8^, ensuring access to safe, fair and meaningful work that supports the well-being of individuals. These principles reinforce the idea that sustainable employability and participatory health are not only public health priorities but also essential for achieving inclusive and resilient labour markets.^8^

The importance of sustainable employability is further underscored by the fact that work participation contributes significantly to an individual’s overall health and well-being,^9 10^ emphasising the need for healthcare services that focus on work ability. This perspective is supported by the WHO’s International Classification of Functioning, Disability and Health, which conceptualises health in terms of functioning and participation, including work participation as a critical domain of life of an individual.^11^ Furthermore, the concept of work ability has also been proposed as a central outcome and organising principle.^12^ It emphasises the dynamic balance between a person’s health, functional capacities and the demands of their work and highlights the role of personal as well as environmental factors in achieving sustainable work participation. Responding effectively to changes in work ability requires coordination between different healthcare professionals, as both medical and contextual factors should be addressed.^13^ This is further supported by the so-called work disability paradigm, which describes work disability not merely as a result of illness or injury but as a complex interaction of personal, workplace, healthcare, compensation and societal factors.^13^ Preventing unnecessary work disability, thus, requires a system-level response that involves multiple stakeholders. A previous scoping review on clinical work-integrating care concluded that both work-related healthcare professionals, such as occupational physicians, and curative care providers should be involved in delivering this work-focused healthcare to support patients’ work participation and socio-economic needs.^14^ This is especially critical for individuals experiencing chronic illnesses or disabilities, who require comprehensive support from healthcare professionals across various disciplines.^5^ Especially, the role of the general practitioners (GPs) alongside occupational health professionals has been recognised to contribute to better work participation, as they act as the first point of contact for navigating between different sectors.^15 16^ Moreover, professionals such as physiotherapists have been found to be of added value in providing work-focused healthcare.^17^ However, those professionals are often not optimally integrated into work-focused healthcare pathways. Multidisciplinary collaboration and communication among professionals, which are critical for delivering effective work-focused healthcare, are often not optimally implemented.^5 18^ However, despite the recognised importance of supporting sustainable employability, patients continue to experience difficulties in returning to work. Previous research has shown that patients with chronic illnesses often feel responsible for coordinating their own care, especially when curative and occupational health professionals provide fragmented care without shared protocols and without a responsible person taking lead of the process such as supervisors.^19^ Patients frequently report a lack of coherent interaction among medical, psychological and occupational health professionals, leading to inconsistent information, confusion and distrust.^5 18^ This fragmented communication often leaves patients to navigate the complexities of work participation on their own, without adequate support.^20^

Multi-professional collaboration has long been recognised as essential for delivering coherent, person-centred care in both clinical care and primary care settings, particularly to ensure collaborative approaches in addressing the biopsychosocial needs of patients managing chronic health conditions.^21^ Previous research has highlighted ways in which multidisciplinary collaboration in clinical rehabilitation can be improved, with tools developed to support knowledge translation and enhance team functioning.^22^ In recent years, the focus of research has expanded to include multiprofessional collaboration across general and occupational healthcare.^23^ Collaboration, however, as described above, work-focused healthcare is broader than occupational healthcare. To date, little is known about the barriers to, and potential improvements in, collaborative approaches within this broader scope, including, for instance, paramedics such as occupational physiotherapists and social insurance physicians.

Previous research has shown that sustainable work participation outcomes cannot be achieved through intervention from one domain only, it being from the curative care or work-focused healthcare domain. ^24^ Improving work participation and return to work (RTW) requires a committed collective effort from all professionals involved.^5 20 24^ Integrated work-focused healthcare requires healthcare system design that addresses the needs of both patients and professionals involved.^5 20 25^ Although previous studies have explored patients’ needs in work-focused healthcare,^5 20^ there remains a gap in understanding how professionals experience collaboration and what they perceive as necessary for improvement. This study addresses that gap by systematically exploring perspectives of healthcare professionals involved in work-focused healthcare. Overall, this study aims to explore the (1) motivations related to interdisciplinary communication and collaboration, (2) barriers in current interdisciplinary communication and collaboration and (3) potential solutions for achieving optimal communication and collaboration across professional domains within work-focused healthcare from the professionals’ perspective. By exploring the motivations, barriers and potential solutions, this study contributes to the development of more integrated strategies to support better work participation and more effective collaborative practice.

## Methods

### Study design and setting

The study employed a qualitative exploratory interview design, utilising a general interview guide approach.^26^ This design was chosen because it is well-suited for exploring complex topics where in-depth insights into experiences, perception and contextual factors are needed. It allowed for structured yet flexible conversations with various professionals involved in work-focused healthcare in the Netherlands to identify motivations, barriers and potential solutions for multi-professional communication and collaboration to promote work participation and improve RTW outcomes.

The Dutch healthcare system is distinctive for its strict separation between treatment and assessment, resulting in a clear division in curative care and social care.^20^ This unique structural principle aims to promote objectivity and impartiality, reducing potential conflicts of interest that could arise if treating physicians were involved in decision-making regarding work capacity, work ability and eligibility for disability benefits. However, this separation of care into distinct silos creates unwanted gaps in care trajectories. Notably, the Gatekeeper Improvement Act (GIA, Wet Verbetering Poortwachter) formalises the limited role of curative care professionals in the work participation and RTW processes. Their main responsibility is to provide medical treatment without directly engaging in the RTW process or making decisions about work capability. Besides the GIA, other legislation such as the Sickness Benefits Act and the Work and Income according to Labour Capacity Act also frame the legal context for social security in the Netherlands. Work-focused healthcare professionals, including among others occupational physicians, social insurance physicians and occupational physiotherapists, are responsible for assessing work capacity, providing RTW guidance, offering support or interventions to promote work ability and advising on workplace adjustments to accommodate health conditions. These professionals also play a crucial role in disability assessment and determining eligibility for social security benefits. Additionally, occupational physicians can refer patients for treatment if work-related health issues are identified. The term ‘work-focused healthcare’ encompasses these responsibilities and represents a healthcare approach that integrates the improvement of work capability and assessment into the management of health conditions. The study was conducted by researchers from Amsterdam University Medical Centre in collaboration with Master’s students Design for Interaction from Delft University of Technology. Amsterdam University Medical Centre led the study design, data analysis and manuscript development, whereas interviews were conducted by the students and supervised by researchers from both Amsterdam University Medical Centre and Delft University of Technology. The reporting of this qualitative study followed the Consolidated Criteria for Reporting Qualitative Research guidelines (online supplemental additional file 1).

### Participants and recruitment

Participants were recruited using maximum variation purposive sampling to ensure a diverse range of perspectives.^27^ Recruitment was conducted via the researchers’ professional networks and through targeted social media outreach on LinkedIn, where posts were shared by the students. The sample included healthcare professionals (GPs) and work-focused healthcare professionals (occupational physicians, social insurance physicians and occupational physiotherapists). These groups of professionals were selected owing to their role in improving work ability and supporting or facilitating RTW and sustainable employability. The division between curative and work-focused healthcare shaped participant selection, as it highlights the need to capture perspectives from both domains. Therefore, GPs were intentionally included from curative care owing to their unique position as the first point of contact in curative care. In the Dutch healthcare system, GPs often remain involved throughout a patient’s care trajectory. Although GPs do not formally lead the RTW process, their role in early identification and patient relationship is essential. Similarly, occupational physiotherapists were chosen because of the growing recognition of the importance of physiotherapists in the work-focused healthcare trajectories.

Additional inclusion criteria were a minimum of 1 year of professional experience in their respective field and active involvement in patient care. The participants were invited to participate via email where information was provided and written consent was sought. The goal was to interview at least five professionals from each of the four professional groups. This target was based on previous qualitative studies in similar domains and aimed to balance diversity with feasibility for in-depth analysis.

### Data collection and analysis

Semi-structured interviews were conducted between February 2021 and June 2021 using a pre-developed interview guide to structure the conversations, allowing for consistency in the main topics covered across interviews while offering flexibility for follow-up questions depending on the response of participants.^27^ The interview guide was developed by the students, and alignment was achieved during presentations by the students before data collection. To ensure robust alignment and standardisation, students presented preliminary versions of their interview guides, which were reviewed with constructive feedback from the first and fourth author (NZ and MM). Additionally, the students conducted simulated interviews before formal data collection to support consistency in interview technique. This process resulted in a single, unified interview guide, which was used by all student teams, facilitating consistency in data collection and analysis. The final version of the guide was approved by the researchers. Questions included the following: Can you describe a situation where collaboration with other professionals involved in work-focused healthcare went well or poorly? What challenges do you encounter when trying to deliver work-focused, patient-centred care? What would help improve communication and collaboration in your daily practice? The following topics were also covered: experiences with perceived barriers in current communication and collaboration with other professionals, motivations regarding multi-professional communication and collaboration and potential solutions on how to achieve optimal multi-professional communication and collaboration. For details, see online supplemental additional file 2. Interviews were conducted via video conferencing due to the COVID-19 pandemic, which restricted personal contact. Interviews lasted approximately 45–60 min. All interviews were audio-recorded and transcribed verbatim. Given the international character of the master’s programme of students who conducted the interviews, interviews were conducted in both English and Dutch, depending on the group composition of students. All participants were informed in advance about the preferred language for the interviews. A minimum of three students conducted each interview, with one student serving as the primary interviewer, whereas the other two provided additional support. Since the interviews were conducted by master’s students and the analysis was performed by the research team, several measures were taken to ensure the transfer of contextual understanding. First, the student teams provided written summaries and oral debriefings for each interview to the research team, highlighting key dynamics and verbal nuances observed during the interview. Second, audio recordings were available to the research team, allowing them to revisit tone and context where necessary. The research team randomly reviewed a subset of audio recordings to check for consistency in delivery and adherence to the interview guide. These measures helped maintain continuity between data collection and analysis, enhancing credibility of the findings.

The goal was to include five professionals per group, as this would offer sufficient diversity to identify key themes. The characteristics of participants (sex and professional group they belonged to) were gathered and analysed, with categorical data presented as frequencies and percentages. Qualitative data were analysed by thematic analysis to identify common themes for each of the three research questions from the interview transcripts.^28^ The analysis combined deductive and inductive elements: data were deductively grouped according to the three research questions, whereas codes and themes were developed inductively from the transcripts. Each transcript was initially coded line by line by the first author (NZ), identifying relevant text fragments and assigning open codes. These codes were grouped into themes based on conceptual relationships. The themes and codes were initially reviewed by the second author (MH), and disagreements were resolved by discussion. To further refine and validate the results, a detailed review of all themes and codes was conducted by the last author (SvdB-V). This multi-step approach enhanced the study’s credibility by incorporating researcher triangulation and strengthening the consistency of theme and code development. In this way, theme development was not linear but iterative, involving repeated returns to the data to check alignment between codes, themes and underlying meaning. Although member checking was not conducted, the findings are supported by illustrative participant quotes to enable thick description and ensure that the reader can evaluate the interpretation.^29^ The results from the various stakeholder groups were analysed collectively, as a joint analysis was expected to yield more informative insights for developing improved collaborative care in the future. Only when the perspectives of the stakeholder groups differed, then they were considered separately. Due to the explorative nature of this study, the goal was not to reach data saturation in the strictest sense but to obtain a broad and representative overview of professional perspectives across the relevant disciplines. Although one student group included additional participants, the other groups did not expand further because of time and feasibility constraints. For the analysis, MAXQDA was used for coding. Member checking of transcripts and findings was not performed as part of the data verification process, and no repeat interviews were carried out. During manuscript preparation, large language models, such as Microsoft Co-pilot, were used to support language formulation and editing. These tools were not used for any content-related analysis or interpretation of the study findings.

### Role of the researchers and ethical considerations

Master’s students who conducted the interviews had limited experience in qualitative interviewing and were not familiar with work-focused healthcare organisation in the Netherlands. They received an informative introductory presentation from the first author (NZ) and were supported by senior researchers (NZ and MM) in shaping the aim, relevance and design of the study. NZ, MH, MM, EC and SvdB-V are experienced qualitative researchers with expertise in occupational healthcare, human-centred design and quality of care improvement.

The study was conducted according to the principles of the Declaration of Helsinki and in accordance with the Medical Research Involving Human Subjects Act (WMO). A non-WMO declaration was obtained from the Medical Ethics Committee of the Amsterdam University Medical Centre (reference number: W20_421 # 20.468). Participants provided written informed consent after receiving an information letter with detailed explanation of the study’s purpose, procedures, and their rights, including confidentiality and the voluntary nature of participation. Participant anonymity was ensured throughout the analysis and reporting phases.

## Results

A total of 22 professionals participated in this study. Characteristics of participants are presented in table 1. The qualitative thematic analysis provided insight into motivations, perceived barriers and identified opportunities for improvement for collaboration between curative care professionals and work-focused healthcare professionals focusing on work participation and RTW processes. This structure was chosen to first illustrate the perceived relevance and urgency of the topic before the specific barriers and solutions. Semi-structured interviews with occupational physicians (n=5), social insurance physicians (n=5), GPs (n=7) and occupational physiotherapists (n=5) revealed three main themes and nine subthemes, characterising the multifaceted motivations, barriers and possible solutions to enhancing collaboration and communication (online supplemental additional file 3).

**Table 1:** Participant characteristics

Characteristic	Frequency (%)
Men	10 (45.5%)
Women	12 (54.5%)
Professional group	
Occupational physician	5 (22.3%)
Occupational physiotherapist	5 (22.3%)
Insurance physician	5 (22.3%)
General practitioner	7 (31.8%)

### Motivations for interdisciplinary communication and collaboration

Professionals across disciplines expressed a shared belief in the importance of improving communication and collaboration in work-focused healthcare. Several professionals, particularly GPs, stated that the ‘delivery of adequate work-focused care’ is especially important ‘for people with chronic illness’ experiencing work participation problems for a longer period. Many participants highlighted that collaboration becomes particularly relevant in complex or stagnating cases, where additional professional perspectives are needed to ‘maximise recovery efforts’. This reflects an understanding that no single professional group possesses all the necessary information or expertise to address the multifactorial determinants of work participation. The professionals highlighted that better collaboration, and thereby more complete information, enables them to ‘contribute to person-centred care’ by ‘minimising divergent medical opinions’ in the care provided between professionals that may arise when professionals work in isolation. Such divergences may not only delay recovery but also confuse patients navigating complex decisions in their RTW process. In this way, professionals believe that they can ‘strive for more patient value’ instead of prioritising the value of healthcare providers or society. The following quote illustrates how professionals conceptualise collaboration as enabling a broader, participation-oriented approach rather than purely biomedical decision making:

*If we abandon this ineffective system and instead focus on how we can help this person— whether they claim to be ill, or claim to be happy managing five children, or happy being a volunteer at whatever—it allows us as physicians to help people find their place in society, not just within the economic framework.* (Insurance physician, female)

This suggests that professionals view interdisciplinary collaboration as a way to shift from institution-centred care, often shaped by siloed responsibilities, formal obligations and reimbursement systems, toward a participation-oriented model. In such a model, collaboration is not only a technical necessity but an ethical commitment to ensuring that care delivery supports the broader social inclusion and functioning of individuals.

### Experienced barriers in current communication and collaboration between professionals

#### Lack of preconditions for effective communication and collaboration

Many participants reported that the lack of basic structural and organisational preconditions hinders effective interdisciplinary communication and collaboration. Although professionals expressed a clear desire for faster and more direct communication, they indicated that current communication channels are fragmented, slow and often informal, which limits timely information exchange and coordinated decision making. One participant specifically mentioned ‘slow and fragmented communication’ to emphasise that more rapid and more direct communication channels are needed. This barrier not only delays mutual updates but also hinders shared decision-making and continuity of care across domains. In addition to the lack of communication infrastructure, participants pointed to a ‘lack of care coordination frameworks’ as a key barrier. Professionals described siloed service provision with no shared platform or structure to support collaborative work. This fragmented organisation was seen as especially problematic in cases requiring integrated support to prevent work disability or facilitate RTW. One GP highlighted:

*Yes, I would strongly advocate for a kind of stepped care model, where the municipality also plays a role. So that there is regional collaboration.* (GP, male)

This perspective illustrates that professionals not only face practical communication challenges but also the lack of clear collaboration frameworks necessary for more coordinated service provision.

#### Lack of timely and proactive triggers for interdisciplinary communication and collaboration

Participants reported that interdisciplinary communication and collaboration are often initiated too late in the care trajectory, which hinders early intervention and coordinated decision-making. Professionals recognised that ‘late initiation of collaboration’ often results in communication occurring predominantly reactively, after work participation problems have escalated or stagnation occurred, rather than at the start of a patient’s care trajectory. As an insurance physician explained the importance of early communication:

*What we observed is that if there is a [health] problem arising and they [other professionals] communicate with us early enough, we can together look for a good solution, which in the end is, of course, the best for the client as well.* (Insurance physician, female)

Furthermore, professionals emphasised the ‘lack of structure to facilitate preventive actions’, noting that interdisciplinary communication is rarely triggered before problems become complex or entrenched. This highlights a systemic barrier where collaboration is embedded as a reactive, rather than proactive, component of care delivery, thereby limiting potential of interdisciplinary approaches to prevent work disability.

#### Lack of shared work-related knowledge and common goals

Many participants identified a lack of shared knowledge regarding their patients’ work-related issues, as well as the absence of common goals in treatment plans as significant barriers to effective collaboration. This disconnect is particularly pronounced between curative care providers and work-focused healthcare professionals, who often operate with fundamentally different frames of reference. Participants from both curative and work-focused healthcare noted that ‘curative care lacks focus on work-related issues’ and that professionals involved in curative care and occupational professionals have ‘different treatment goals’. Curative care professionals typically aim for symptom resolution and disease management, whereas work-focused care professionals focus on functional recovery and reintegration into work. Moreover, participants expressed that they are somewhat ‘reluctant to share information from curative care to work-related care’ as illustrated by a quote from a GP, explaining concerns about potential legal consequences of written communication:

*Well, yes, when you put something in writing, you become a bit cautious, right? Because if there ever comes a lawsuit or something, what’s written in black and white can be used against you, as they say. If you’ve said things about a patient, it’s a bit harder when it’s verbal, so then I find it easier to speak. You can also assess how the occupational physicians or insurers respond.* (GP, male)

This reflects a preference for verbal communication over written documentation when interacting across domains. The preference for verbal communication stems not only from legal caution but also reflects a lack of formalised, trusted communication structures. Reliance on verbal exchanges contributes to fragmented information sharing and hinders the development of shared goals. Such conversations are often sporadic, remain undocumented and depend on personal rapport, making them an unreliable foundation for coordinated care.

In addition, the *‘*perception that occupational physicians are not independent’ is mentioned as a barrier by both curative and work-focused healthcare professionals, limiting the willingness to share information. Occupational physicians are often viewed as acting primarily in the interest of employers rather than patients. This perception undermines trust and further reduces professionals’ willingness for open communication and information sharing, reinforcing siloed practices.

Finally, several participants described ‘insufficient insight into each other’s professional roles’, which may hinder them from collaborating with each other. Without a clear understanding of what the other profession contributes, professionals may find it difficult to establish a shared language, mutual trust and aligned objectives. Without sufficient insight into each other’s professional roles and responsibilities, collaboration remains superficial, making integrated, patient-centred care challenging to achieve.

#### High workload

Participants reported that communication and collaboration between professionals is not only time-intensive but also embedded within a system that lacks efficiency and alignment. Participants noted that they experience communication and collaboration as ‘time-intensive’, contributing to significant frustration in professional interaction, particularly due to the time lag in obtaining formal authorisations and the information requested from curative healthcare professionals. Professionals explained that such processes can take several weeks, delaying decision-making and reducing perceived benefits of collaboration:

*[…] the process of sending an authorization and then getting it back—if it’s fast, you’ll have it back in one and a half to two weeks. Then you still need to get the information from the specialist or the general practitioner, who also need to make time and space for it.* (Occupational physician, male)

Beyond systemic delays, participants described the frequency and volume of formal requests for written information as burdensome. They expressed that they are tired of the ‘number of requests for written medical information’. Participants, especially GPs, noted that the overwhelming number of requests for sharing information was experienced as exhausting, which led to a burdensome experience. Moreover, the professionals highlighted that the ‘busy schedules limit peer consultation’, creating a burden in balancing the administrative tasks of collaboration with their other clinical or guiding and supporting responsibilities. The combination of rigid formal processes and insufficient structural support for informal contact undermines the feasibility of timely and meaningful interaction across domains. An occupational physician expressed the difficulty in establishing contact with other specialists in the hospital, stating:

*There’s very little contact with other specialties in the hospital, mainly because it’s difficult to connect. Of course, they're busy, we're busy, and practically speaking, there’s never enough time.* (Occupational physician, female)

This illustrates how heavy workloads, lack of protected time and inefficient procedural infrastructures create interlocking barriers that constrain collaborative capacity. Although professionals may be motivated to engage with colleagues across domains, the current system provides neither the time nor the structural support necessary to do so effectively.

#### Limiting rules and regulations

Regulatory constraints were mentioned as significantly limiting the opportunity to communicate and collaborate with other professionals. Some participants noted the ‘fragmentation between healthcare and social welfare systems’ as a barrier to collaboration. The disparity in funding sources could complicate collaboration, as the healthcare system and social welfare system are separated in the Netherlands. Moreover, the ‘need for informed consent’ by the patients was noted as a barrier, as it imposes legal and ethical obligations that must be navigated and in some cases may prevent possibilities for communication.

According to current Dutch guidelines for medical information exchange between curative healthcare and occupational healthcare professionals, the disclosure of medical information requires targeted consent from the patient, specifying both the purpose of the request and the type of information to be shared. In practice, this requirement is frequently interpreted as necessitating written consent. This interpretation of the legal criteria for informed consent encourages risk-avoidant behaviour, with professionals reluctant to rely on informal or verbal consent. For example, an insurance physician described:

*With the medical doctor in the hospital, I cannot communicate and I’m not allowed to, because I have to wait for the informed consent of the patient.* (Insurance physician, male)

The absence of user-friendly processes for recording consent across systems further reinforces reluctance to collaborate and communicate more with other professionals.

Professionals experienced that there are ‘strict regulations for exchange of medical information’, illustrating the frustration with current procedural rules and regulations that do not accommodate smooth communication and collaboration. Taken together, these findings highlight the need for greater legal clarity regarding consent requirements, alongside practical tools and infrastructures that support ethical, efficient information exchange, while safeguarding patient privacy.

#### Insufficient facilities to communicate

The lack of effective and efficient communication tools was experienced as a critical barrier to interdisciplinary collaboration. Existing systems do not give room for timely and direct communication, as professionals mentioned ‘no direct communication’ as an important barrier. This lack of infrastructure can lead to delays and practical inaccessibility. In the current system and facilities, professionals expressed the lack of direct communication lines between work-focused healthcare professionals and curative care professionals. Thus, making communication rely heavily on patients or ad hoc personal networks.

The participants also described the current system as two separate worlds where they are ‘not able to find other professionals’ which impedes their possibility to contact each other and limits their opportunity to build a relationship with other professionals:

*It can be useful to speak to each other. But before you even know who the occupational physician is at which company, you’re already so drained by the effort that you think, ‘Well, just let the patient sort it out themselves’.* (GP, female)

Moreover, the ‘market mechanism in digital solutions within healthcare’ was frequently mentioned as a barrier by the professionals. The current digital systems in the Netherlands were described as incompatible across domains, meaning that professionals from curative care and work-focused care cannot access or share patient information through a shared electronic health record. Several participants expressed concerns that the market mechanism in digital health solutions has led to a patchwork of systems that do not interface with each other, limiting shared access to patient information and care plans. They also mentioned experiencing ‘deficiencies in secure data exchange’, which are legally required, that hinder their willingness to exchange data, as one insurance physician mentioned that a system to exchange data in a secure manner does not work half of the time. These examples reflect how technical and organisational deficiencies in communication infrastructure contribute to a sense of professional isolation and inefficiency, ultimately limiting coordinated care.

### Solutions to reach optimal communication and collaboration

#### Insight into each other’s role

A recurring theme among participants was that a lack of mutual understanding of professional roles impedes effective collaboration. Professionals expressed that building insight into each other’s expertise and responsibilities and recognising each other’s contributions foster not only better communication but also trust and accountability. Therefore, the professionals proposed the solution to ‘learn about the other’s role’ supporting the ‘development of a personal relationship’:

*It’s important, I think, what we are trying to achieve in our network is that you know each other and understand what you can expect from one another, and then you know that you’re on the same page and that you can hold each other accountable because I mean, I’m not infallible either, so if I miss something and a physiotherapist tells me, ‘Hey, that’s not right,’ and I know him and how he works, then I’m more likely to take it seriously. So knowing each other is really important in the community.* (GP, male)

Knowing how another professional operates leads to stronger engagement and responsiveness in interdisciplinary dialogue. Rather than viewing collaboration as a bureaucratic task, professionals described it as a relationship-based process grounded in recognition of each other’s role and contribution and reciprocal respect. Several suggestions were made to strengthen this shared understanding, including structured opportunities for interaction such as joint meetings, delivering lectures to one another, or organising interdisciplinary education.

*So, we would progressively visit various meetings of general practitioner groups and ask for half an hour of their time to, together with a colleague, so two occupational physicians, give a sort of lecture about what the occupational physician does and what we would like to achieve in collaboration.* (Occupational physician, female)

These strategies highlight that improved role clarity does not merely enhance technical coordination but also reduces professional hierarchy, increases perceived legitimacy and strengthens appreciation of each other’s value in the care trajectory, all of which are essential for meaningful communication and integrated care.

#### Clearer coordinated care

To address current inefficiencies in communication and collaboration, professionals proposed solutions that centred on optimising care coordination and streamlining task distribution. A key element mentioned was the need for ‘clear, centralised coordination of care’, preferably led by a professional who maintains oversight and ensures alignment across disciplines. The coordination should be managed by a professional who is central to all other professionals involved. GPs or occupational physicians were most frequently identified as suitable coordinators owed to their broad involvement in the patient’s care trajectory. This call for central coordination reflects a deeper frustration with fragmented responsibilities and unclear professional boundaries. Professionals argued that designated care coordination would prevent duplication, promote consistent messaging to patients and facilitate continuity of care across settings. In addition to coordination, some professionals indicated that more efficient collaboration and communication could be stimulated by *‘*the delegation of tasks’ among the involved professionals, which is particularly important in response to workforce shortage across both domains. For example, occupational physiotherapists proposed to take on specific tasks under the supervision of occupational physicians, thereby reducing pressure on scarce resources and enhancing team-based care delivery:

*I think that we could actually serve as good extension or whatever for what the occupational physicians are doing now, as there are too few of them. There are too many tasks, creating a need for task delegation. We could really effectively fill that role, in managing physical strain, under the supervision of the occupational physician. That’s something that, I think, we have not fully recognized yet.* (Occupational physiotherapist, male)

In addition, professionals indicated a need ‘to consult with other professionals in real life or by telephone’ for effective communication. This was seen as essential to reduce delays, enable timely clinical decision-making and support mutual understanding. These suggestions indicate a shared awareness that improving relational and structural efficiency in communication processes is vital to delivering integrated, person-centred care.

#### Digital care solutions for safe and efficient information exchange

Professionals proposed several digital innovations to overcome the current barriers posed by legal, infrastructural and organisational constraints on interdisciplinary communication. A recurring suggestion was to improve how patient consent is obtained and documented. Several professionals proposed the development of ‘digital solutions to streamline informed consent’, reducing reliance on paper forms or repeated verbal requests.

*Have you ever considered how nice it would be if, with your insurance card, perhaps with a chip on it, you could be at a doctor’s office and simply place your card on a device or nowadays use your phone to grant permission by entering a PIN or something similar? Then you’d say, ‘I give permission for you as a doctor to share information with so-and-so’, and then it’s all arranged. Essential parts of the medical dossier would be shared because we don’t need to know everything, right? […] This would make it easier to consult.* (Occupational physician, male)

Beyond consent mechanisms, many professionals stressed the need for a ‘digital cross-sector platform’ to support interdisciplinary communication in work-focused healthcare. Such a platform should include a ‘digital directory of contact details’ of involved professionals*,* allowing for tracking of information requests and support clarity on roles and responsibilities, enabling users to know who is involved, how to reach them and who is coordinating care. As one participant explained:

*Having every occupational physician listed in [existing curative care sector platform] would obviously make things completely unmanageable. But if there was a separate system, perhaps something like ‘Care portal work’ or similar, then the information that we gather in the consultation room, I would want to report that in the same system and forward it to the occupational physician, saying, ‘Today I saw one of your employees in connection with stress-related complaints possibly due to the work situation,’ or something to that effect.* (GP, female)

Furthermore, professionals also emphasised that digital tools should ‘enable person-centredness’, with ‘role-specific access rights’ that ensure each professional only sees information relevant to their function. Suggestions included to ‘enable patient’s ownership of their personal health information’, allowing them to decide what is shared, with whom and when. These digital innovations were viewed not only as technical fixes but as structural enablers of trust, efficiency and shared responsibility across curative and work-focused healthcare.

## Discussion

In this qualitative exploratory interview study, we gained insight into (1) motivations regarding multi-professional communication and collaboration, (2) experienced barriers in the current multi-professional communication and collaboration and (3) possible solutions on how to reach optimal multi-professional communication and collaboration regarding work-focused healthcare from professionals’ perspective. Our findings provide a more refined and detailed understanding of professionals’ perspective and highlight how context-specific and system-related factors shape communication and collaboration across domains. This study highlighted the need for systems that enable direct and efficient communication between professionals. A central digital platform was identified as a pivotal solution to enhance information exchange and foster relationships between curative and work-focused healthcare professionals. Integrated approaches and technical solutions to bridge practical gaps were considered essential. In this study, professionals emphasised that effective work-focused healthcare depends on timely, proactive and person-centred collaboration between curative and work-focused professionals. Professionals emphasised that collaboration is particularly valuable at key moments, early in the care trajectory, during diagnostic uncertainty, when patients receive conflicting advice, or when reintegration stagnates. These moments were seen as critical to avoid delay, provide consistent advice and ensure continuity of care. Similarly, previous research stressed the importance of early interdisciplinary collaboration to support sustainable work participation, especially in complex or chronic cases.^5 17 18^ Collaboration should be guided by clear coordination structures and mutual understanding of roles to ensure coherent decision-making.^18 30 31^ However, professionals in our study reported several barriers to such multi-professional collaboration. Lack of shared knowledge, differing professional perspectives, limited understanding of one another’s roles and insufficient information about the patient’s context were identified as key barriers. These findings align with previous studies that highlight how differing frames of reference and siloed knowledge systems can restrict effective interdisciplinary collaboration.^5 17 18^ In this study, time constraints, restrictive privacy regulations and fragmented communication infrastructure were additional structural barriers that reinforce these misalignments.

To overcome these challenges, professionals proposed various solutions aimed at improving both the process and structure of collaboration. One key solution was clearer coordination of care, supported by a central coordinating professional, ideally a GP or occupational physician, to streamline communication and align tasks and responsibilities across care providers and domains. This central role could provide the basis for enhanced efficiency and clarity. However, previous research highlighted that financial constraints and insufficient authority for such a coordinating role present significant challenges.^32^ Another barrier is the lack of time, which both our study and earlier research have identified as key barriers, particularly for occupational physicians and GPs,^33 34^ partly because these tasks are not included in the current care agreements and the corresponding compensation for performed care tasks. This challenge is further compounded by a shortage of occupational physicians, increasing pressure on the already limited time available for collaboration.^35 36^ Furthermore, the lack of visibility and integration of occupational physicians within curative care settings aggravated the issue, as they often work in isolation rather than as part of an integrated, multiprofessional team.^36^ Overcoming these barriers requires structural reforms in care organisation and adjustments to rules and regulations.

Professionals also strongly advocated for the development of a digital platform tailored to work-focused healthcare. In the Netherlands, information exchange between work-focused and curative healthcare professionals is strictly regulated and requires explicit patient consent.^37^ To facilitate efficient and seamless information sharing, clear multi-professional guidelines and protocols are essential. However, their successful use depends not only on design but also on successful implementation. Prior research within Dutch occupational healthcare has demonstrated that guidelines are often underutilised by occupational physicians. This can be attributed to several factors, including a lack of awareness of the guidelines’ existence, a perceived absence of added value, practical barriers in their application and insufficient stringency.^38^ To overcome these challenges, particularly when developing future guidelines that promote interdisciplinary collaboration, it is essential to actively involve all relevant professionals and clearly demonstrate the practical benefits of such guidelines.^39^ The study participants suggested that digital consent solutions could simplify the process, enabling smoother information exchange while respecting patient privacy. This is in line with the results of a previous study that explored the requirements for an electronic handover system between psychotherapists and occupational health professionals.^40^ They also highlighted, while promising, that such systems must ensure data security, user-friendly design and integration with existing workflows.^40^ Similarly, a previous study found that effective digital communication tools in occupational health must be tailored to enhance usability and accessibility.^41^ Patient portals may be another solution to limit fragmentation and delay in information exchange.^42^ However, implementing such portals across complex, interdependent networks presents substantial challenges, particularly in ensuring regulatory compliance and interoperability between systems.^42^ Hence, the design and implementation of such a solution will require co-creation with stakeholders, legal clarity and iterative development and testing to ensure successful uptake and effectiveness.

Additionally, this study suggests that clear role definitions and mutual understanding among professionals are essential for fostering effective communication and collaboration. A lack of clarity regarding each other’s roles and contributions in work-focused healthcare often hinders access to the necessary professionals and limits meaningful engagement.^43 44^ Building personal relationships is therefore crucial in establishing more communication and collaboration.^43 45^ Interprofessional education may offer a promising solution to bridge these gaps and strengthen collaborative practice.^46 47^

Improving the quality of care and collaboration between professionals also demands consideration of patients’ perspectives.^48^ A previous qualitative evidence synthesis highlighted a significant communication gap between involved professionals in work-focused healthcare, leading to inconsistent information and discrepancies in what is communicated towards the patient.^18^ Patients particularly seek a clear and continuous process including coherent and constructive collaboration between professionals involved in work-focused healthcare.^18^ In this context, patients have recommended that occupational health professionals should be integrated into multidisciplinary clinical teams, potentially reducing the perceived conflicting involvement with employer interests.^18 20 49^ The added value of forming multidisciplinary teams within curative care and including work-focused healthcare professionals would facilitate a more holistic treatment approach.^49^ This integration would ensure that treatment plans are more aligned considering the patient’s needs.^20 49^

Differences in emphasis and priorities emerged between professional groups. For example, occupational physicians and insurance physicians more frequently highlighted regulatory constraints and barriers to information sharing, likely due to the strict legal frameworks they must adhere to.^49^ In contrast, GPs primarily emphasised time constraints and uncertainty about their role in work-focused care, reflecting their broader yet less formalised involvement in RTW processes. These variations suggest that the challenges professionals face, as well as the solutions they propose, are closely tied to their structural positions, responsibilities and level of integration within current work-focused healthcare practice.^50^

Although this study highlighted a range of practical and professional-level solutions, regulatory constraints would also need to be addressed to achieve systemic improvement. The absence of system-level solutions in this study may reflect a perceived lack of mandate or influence over policy among professionals. However, a previous study emphasised that work disability is shaped by a complex interplay between personal, clinical, workplace and systemic factors including legislative structures, requiring integrated solutions beyond the professional level.^13 24^ The limited attention to system-level solutions in our findings highlights the importance of involving policymakers and other stakeholders in future co-creation processes to support sustainable implementation of interdisciplinary practice within integrated work-focused healthcare.

Beyond the focus on collaboration and communication between professionals in work-focused healthcare, it is important to acknowledge that improving work participation and preventing work disability requires coordinated efforts that extend beyond professionals and stakeholders alone.^24^ Work disability results from a complex interaction of personal, workplace, healthcare, compensation and societal factors. Therefore, sustainable solutions should also take into account the engagement of policymakers, employers, the social environment of a person and other institutional stakeholders responsible for shaping this complex system.^13 24^ This makes an integrated care model even more relevant, as they should be embedded within supportive organisational and regulatory environments that can address the complexities of work disability. A previous study showed that certain medical or non-medical interventions can be effective in tackling work participation outcomes; however, their implementation remains challenging.^24^ Therefore, future strategies should involve all relevant stakeholders beyond the work-focused healthcare professionals.^24^

### Methodological considerations

Although this study provides valuable insights to contribute to the development of more integrated work-focused healthcare, several limitations should be acknowledged. First, inter-interviewer variability may have influenced the data, as different students served as the primary interviewer across sessions. This variability was minimised by maintaining a consistent main research question and using interview guides that were rigorously checked and approved by the senior researchers. The interviewers also received supervised instructions to promote consistency in interview conduct. Additionally, the full transcripts were reviewed and thematically analysed by the first and second authors, both experienced in qualitative research, providing an additional consistency check to ensure coherence in the analysis across interviews. Nonetheless, subtle differences in tone or follow-up questioning may have influenced the depth or focus of some responses. Second, the results were also limited by linguistic uniformity of the interviews. Although some interviews were conducted in English, not all participants were native English speakers, which might have restricted the expression of nuanced views. Participants were informed of the interview language beforehand, allowing them to prepare adequately and ensuring that they could express themselves effectively in either Dutch or English, which would minimise any significant barriers to communication due to language constraints. Nonetheless, some professional or contextual nuances may have been lost. Future studies may benefit from using fully language-matched interviewers or offering participants the opportunity to review translated summaries. Third, while combining views from different professional groups strengthens the analysis by providing a comprehensive overview, this approach could potentially obscure unique perspectives specific to individual groups. Nonetheless, considering the perspectives of all professionals involved is crucial for achieving interdisciplinary work-focused healthcare practice. This study applied thematic coding across professional groups; however, future research could benefit from facilitated discussions among these professionals to foster consensus on actionable solutions. Social desirability bias may also have influenced the findings. Topics, such as collaboration, communication and professional roles, can be potentially sensitive topics. Despite efforts to emphasise anonymity and neutrality, professionals may have provided more socially acceptable responses rather than fully candid reflections, presenting their own roles or responsibilities in a more favourable light. Fourth, a further limitation concerns the sample size and thematic saturation. Although the exploratory approach allowed for a broad and inclusive set of perspectives across multiple professional groups, it may have come at the expense of deeper exploration within each group. As a result, certain aspects specific to particular professional roles may have remained underexplored. Although few new insights emerged in the final interviews per group, the absence of a formal saturation assessment may limit the extent of thematic completeness. Future research could adopt an approach driven by saturation or include a larger sample size per professional group to ensure both breadth and depth of perspectives. In addition, although this study included key professionals from both curative and work-focused healthcare, it may not fully capture the perspectives of all relevant stakeholders involved in work-focused healthcare, such as psychologists, medical specialists, nursing staff or employers. This selection of the four groups was based on their central role in decision-making and coordination within the Dutch system, as well as feasibility considerations. Nevertheless, future research may consider including a broader range of professionals to further enrich the understanding of interdisciplinary collaboration and communication in work-focused healthcare. Finally, the Dutch context may limit transferability of the research results. The separation between curative and work-focused healthcare domains, role of insurance physicians and national consent regulations is specific to the Dutch system. However, many of the reported barriers, such as time constraints, lack of communication tools and unclear responsibilities, are also relevant in an international context. Future research may explore how the current barriers in the Dutch system play a role in other national contexts.

### Implications for practice and future research

Future research should focus on developing multidisciplinary guidelines that address barriers such as inefficient communication tools and unclear role definitions. Evaluating these guidelines in real-world settings can ensure they meet professional needs and foster collaboration. Investigating systemic and regulatory differences in healthcare and social welfare systems may also inform more effective collaborative approaches.

To translate the findings of this study into practice, proposed solutions such as digital platforms, central coordination roles and interprofessional guidelines should be embedded within existing workflows and systems. This requires not only technological infrastructure and interoperability but also alignment with legal and regulatory frameworks, compliance with privacy and data protection standards, ensuring professional role clarity to avoid overlap or ambiguity in responsibilities. Future research on implementation strategies, including facilitation, involvement of end-users, training and feedback mechanisms, can support successful and sustainable uptake. Moreover, co-creation with stakeholders at multiple levels, including policymakers and institutional leaders, is essential to ensure that solutions are not only feasible but also scalable and aligned with broader system goals.^51 52^

Despite the challenges identified in this study, such as time constraints, unclear role definition and lack of communication tools reported in other healthcare sectors,^53^,^55^ this study contributes to the literature by specifically contextualising these issues within work-focused healthcare. It offers a domain-specific perspective on perceived barriers and needs across multiple professional groups and highlights a unique necessity of integrating traditionally fragmented care domains. This contextualisation helps to adapt existing insights on interprofessional collaboration to the RTW context and illustrates clear starting points for structural improvements.

The findings are consistent with broader research on interprofessional collaboration, which has consistently shown that successful collaboration hinges on shared goals, mutual respect, role clarity and robust information exchange.^56^,^58^ However, this study extends this body of knowledge by highlighting how these foundational elements are particularly challenging at the intersection of curative and work-related care, where responsibilities, legal frameworks and information exchange are structurally separated. Moreover, although previous studies have explored facilitators of interprofessional teamwork in primary care^59^ or community-based services,^60^ few have investigated the unique collaboration challenges that emerge in cross-domain care delivery, with different financing, accountability and consent structures, as is the case in Dutch work-focused healthcare.

Establishing common guidelines and a shared digital platform can facilitate easier information exchange. However, the feasibility of such a digital platform depends on its alignment with existing legislative frameworks, interoperability with current systems and acceptance among professionals. These legal, technical and organisational constraints may pose significant barriers that must be considered in the design and implementation of a digital solution. Multidisciplinary training programmes to enhance role understanding and reduce misconceptions can strengthen collaboration. Implementing these solutions could create effective multidisciplinary teams, improving work participation outcomes and patient satisfaction.

## Conclusion

Enhanced multi-professional collaboration in work-focused healthcare requires the implementation of streamlined communication processes and the integration of comprehensive, patient-centred approaches. This study revealed the reasons, contexts and timing for when optimal collaboration and communication add value. However, the identified barriers in current interdisciplinary communication and collaboration indicate that there is still significant potential for improvement. In the future, efforts should focus on the suggested solutions. Future research should investigate guidelines to facilitate clearer communication lines and role definitions among all professionals involved.

## Supplementary material

10.1136/bmjopen-2025-103881 online supplemental file 1

10.1136/bmjopen-2025-103881 online supplemental file 2

10.1136/bmjopen-2025-103881 online supplemental file 3

## Data Availability

Data are available upon reasonable request.
