# Supplementary material for: Exploring motivations, barriers and solutions for interdisciplinary practice in work-focused healthcare: a qualitative study among Dutch healthcare professionals
Source: BMJ Open. 2026 Mar 19;16(3):e103881. doi: 10.1136/bmjopen-2025-103881 (PMC13007094; doi:10.1136/bmjopen-2025-103881)
Supplement: online supplemental file 2 [file bmjopen-16-3-s002.docx]

Additional file 2. Interview topic guide.

General Interview Guide (all professionals)

1. Introduction
   1. Could you briefly introduce yourself and your current role?
   2. How long have you been working in this profession?
   3. What motivated you to enter this field?
   4. What does a typical day in your role look like?
2. Current practice and roles in work-focused healthcare
   1. What is your role in work-focused healthcare?
   2. Which types of patients do you typically see in this context?
   3. In what way does your work relate to supporting patients’ ability to participate in work?
   4. Can you describe a typical patient journey in which you are involved?
3. Communication and collaboration
   1. Can you describe a situation where collaboration with other professionals involved in work-focused healthcare went well?
   2. Can you describe a situation where collaboration did not go well?
   3. With which other healthcare professionals (e.g. GPs, occupational physicians, insurance physicians, physiotherapists) do you collaborate?
   4. How does this collaboration typically take place (e.g. phone, email, digital systems)?
   5. What challenges do you encounter when trying to deliver work-focused, patient-centred care?
   6. Are there any specific professional groups that you would like to collaborate with more?
4. Barriers and needs
   1. What do you see as the main barriers to good communication and collaboration in work-focused healthcare?
   2. How do legal, organisational, or practical factors affect your ability to collaborate with others?
   3. Do you feel your role is sufficiently recognised or understood by other professionals?
   4. In your experience, are there any misunderstandings or assumptions about your profession that hinder collaboration?
   5. How do patients perceive your role in relation to other healthcare professionals?
5. Potential solutions and improvements
   1. What would help improve communication and collaboration in your daily practice?
   2. What tools or systems (e.g. guidelines, digital platforms) would make collaboration easier?
   3. If you could change one thing in the current system to improve collaboration, what would it be?
