# Supplementary material for: Exploring motivations, barriers and solutions for interdisciplinary practice in work-focused healthcare: a qualitative study among Dutch healthcare professionals
Source: BMJ Open. 2026 Mar 19;16(3):e103881. doi: 10.1136/bmjopen-2025-103881 (PMC13007094; doi:10.1136/bmjopen-2025-103881)
Supplement: online supplemental file 3 [file bmjopen-16-3-s003.docx]

Additional file 3. Results of the interviews on the needs related to interdisciplinary communication and collaboration, barriers in current interdisciplinary communication and collaboration, and potential solutions for achieving optimal interdisciplinary communication and collaboration.

| **Category** | **Theme** | **Representative quote** |
| --- | --- | --- |
|  | | |
| Motivation for interdisciplinary communication and collaboration | Delivery of adequate work-focused care for people with chronic illness | “You have to consider that an employer does not need to know if someone had a bladder infection or received an unpleasant diagnosis from a dermatologist. In that sense, it's absolutely not relevant. Even if it's about a miscarriage that necessitates a few days off, I still believe that's not something I should communicate to an employer. However, there are other issues that might be relevant, like someone with chronic knee pain who is still expected to go up and down several floors. Those are the kinds of things I would want to communicate, suggesting that perhaps the occupational physician should also consult with the patient to assess their problems and see if there's a different role within the company that could accommodate their condition.” (GP, female) |
|  | Maximizing recovery efforts | “But actually, at the moment when recovery stalls, right? Or when it appears that someone might not be able to return to their own work due to illness or impairment? Yes, then you definitely need to consult to ensure clarity and confirm if this is indeed the maximum achievable, to have your files in order, but also to be able to discuss thoroughly if we have exhausted all options to achieve the maximum treatment outcome.” (Occupational physician, male) |
|  | Contribute to person-centered care | “I tried to form a good understanding of the client—what kind of person he is, what his social network is like, and what works and doesn't work for him in terms of his mental health. I think this is pretty useful information for physicians in hospitals or for GPs to work with the client and help improve their condition. Maybe more effectively, because they understand what motivates the person or any other relevant factors. My final conclusion, which I would like to send to the GP so that he can expand on it, is something we don't yet do.” (Insurance physician, male) |
|  | Minimizing divergent medical opinions | “Actually, especially at times when, for example, an insurance physician comes to a very different conclusion than I did. For instance, if my assessment was that someone is not able to work or only marginally so, and the insurance physician, after their examination, concludes that the person is quite capable of working, then there's a discrepancy. I would find it logical at that moment for the insurance physician to call me and say, 'That's strange, I've come to a completely different conclusion. Did I miss something, or did you miss something?' So that we can then discuss it together.” (Occupational physician, female) |
|  | Strive for patient value | “Well, it is somewhat all about money, but that does create pressure. But proposals for better collaboration could alleviate that pressure.” (Occupational physician, female) |
| **Experienced barriers in the current communication and collaboration between professionals** | | |
| 1. Absence of preconditions for effective communication and collaboration | Slow and fragmented communication | “There is a need for fast, easy, and direct communication.” (GP, male) |
|  | Lack of care coordination frameworks | “What I would ideally like is for us to have a sort of help desk in [name town] or, say, per municipality. You wouldn’t need to start directly with your occupational physician, but there would be a clear procedure in place for when things get more complicated, allowing you to seek advice from an occupational physician. And, importantly, from a labour expert as well, which we also have access to.” (GP, male) |
| 2. Lack of timely engagement and proactive triggers for interdisciplinary communication and collaboration | Late initiation of collaboration | “I definitely think in the period between zero and one year. But also, I think, after that first year, particularly when it is recognized that with aids, for example, the workplace can be adjusted or perhaps even before the question [illness or disability] arises.” (Occupational physiotherapist, female) |
|  | Lack of structure to facilitate preventive actions | “If you communicate beforehand, you solve the [health] problem before it actually arises, so that's really good.” (Insurance physician, female) |
| 3. Lack of shared knowledge and common goals | Curative care lacks focus on work-related issues | “Also, because for them [curative care professional], work is not important. Their focus is on curing someone, so they say, 'Okay, this is the treatment protocol, right? You get this medication, or you get this radiation treatment, or you get this CT scan or whatever diagnostics.' That's their perspective, and often, they are only concerned with their own medical specialty.” (Occupational physician, female) |
|  | Different treatment goals | “Yes, [the communication with other specialists is] much better, because we all have the same goal, which is not to get back to work as quickly as possible, but to help this patient with their complaints. So, naturally, our perspectives are aligned in the same direction.” (GP, male) |
|  | Reluctant to share information from curative care to work-related care | “Because it's written down, the physicians are very defensive about what they write to me regarding the client's health, and sometimes they are even hostile towards us. This is because we do things the clients do not like.” (Insurance physician, female) |
|  | Perception that occupational physicians are not independent | “Sometimes there is a perception that because the occupational physician is hired and paid by the employer, it follows the adage 'he who pays the piper calls the tune.' Well, I always draw the simple comparison that, just as health insurance companies do not dictate the treatment you receive when visiting your GP or medical specialist, who are also funded by commercial organizations. Iit's really about how we position ourselves and communication about it, which is really super important. And I don't know how far your research has progressed, but if you could also offer advice to our profession on this matters, regarding collaboration with other professionals, because there are sometimes assumptions made in that area as well.” (Occupational physician, male) |
|  | Insufficient insight into each other’s professional roles | “There is insufficient knowledge regarding the work of the occupational physician and moreover, what happens with the shared information? Can this have consequences? They [curative care professionals] lack an understanding of what happens with my [the occupational physician’s] information. I think that is also an important consideration.” (Occupational physician, male) |
| 4. High workload | Time-intensive | “I do think that improvement is possible because ultimately it's about speed. It often takes a lot of time and effort to retrieve information, but also in terms of convenience.” (Occupational physician, male) |
|  | Number of requests for written medical information | “Honestly, I often find it burdensome. Honestly, because then you receive a letter, and it's often from insurance physicians, not just occupational physicians, but also from social insurance physicians at the Social Security Agency, for example. They ask about the diagnoses, what the [medical] conditions are, why someone can't work, what the prognosis is, you know, those kinds of questions. And those letters, I actually never answer them with pleasure. Honestly, it just feels like an additional burden.” (GP, male) |
|  | Busy schedules limit peer consutlations | “[…] because we all have our own schedules, it's difficult to reach someone by telephone.” (Insurance physician, female) |
| 5. Limiting rules and regulations | Fragmentation between healthcare and social welfare systems | “Well, it [communication with other professionals] is all so complicated. Officially, you are not even allowed to have contact [with other professionals].” (Occupational physician, male) |
|  | Need for informed consent | “As general practitioners, we often face challenges due to lack of sufficient insight or influence over certain matters. For example, when an occupational physician contacts us to inquire a patient has visited us for stress-related complaints, we can only confirm or deny this information, andt always with the patient's permission. This is why we need their consent.” (GP, female) |
|  | Strict regulations for exchange of medical information | “The collaboration between occupational and insurance physicians is, frankly, abysmal—it just doesn't exist. The insurance physician is positioned as a kind of judge over the work that the occupational physician has done. And I find that a very strange position to be in. So, I think that there, that collaboration, it just isn't there and that's very odd.” (Occupational physician, female) |
| 6. Insufficient facilities to communicate | No direct communication | “But those [letters] are almost always just standard forms, almost pre-printed, with a signature on them. So, that doesn't really feel like genuine communication with each other.” (GP, female) |
|  | Not able to find other professionals | “So, when you deal with specialists, they are usually the same ones. But if you live in [city name], you already have four hospitals to choose from. So then, you don't build a collaborative relationship, and with occupational physicians, it's even more difficult because on average you only share about three patients with an occupational physician, I believe. If you have large employers, […] then you could build a collaborative relationship with them. But the biggest problems are actually with the smaller employers, the small and medium-sized enterprises.” (GP, male) |
|  | Market mechanism in digital solutions within healthcare | “I always find it difficult when companies, especially those in the service sector of healthcare, aim to profit from it. There's always a bit of—well, reasonableness and fairness involved, you know? People naturally want to make the most of the situation, but they shouldn't make too much money or dominate the market position excessively. So, it's always a bit of a balance, really.” (GP, female) |
|  | Deficiencies in secure data exchange | “We now have systems that allow us to email physicians. However, we also know that some physicians are not accustomed to using the server, and even some of us have problems with it—it works half of the time, and the other half it does not. Additionally, it is an encryption program, so the information sometimes gets jumbled while being sent over. If I know the server [the secure e-mail program] is having issues on a particular day, I might wait a day and try again, but otherwise, I won't wait any longer and will revert to the old methods. If the server works perfectly, we'll use email, but for now, we're sort of in a transition phase.” (Insurance physician, female) |
| **Solutions to reach optimal communication and collaboration** | | |
| 1. Insight in each other’s role | Learn about the other's role | “Having knowledge of what you and the others [other professionals] do and respecting each other’s field, right? Just like in a relationship. That’s the only way you can achieve this [improved communication].” (Occupational physician, female) |
|  | Develop a personal relationship | “[…] the moment you know your colleagues, if I have a general physician here in [name city], where I work, from whom I see a lot of patients, if I speak to that general physician in one of these sessions, I have no problems giving him either my telephone number or my email address. So, if he has a question, please write to me, let me know. I'm not too lazy to answer; just if I don't know you have a problem with me or there is a question, I can't answer it. So, you just have to extend the hand and say, 'Okay, please come, let me know if there's something, and we'll talk about it.” (Insurance physician, female) |
|  |  |  |
|  |  |  |
|  |  |  |
| 2. Clearer coordinated care | Clear, centralised coordination of care | “[…] if other disciplines are involved, you should go through your client and possibly the occupational physician, because they often act as a spider in the web.” (Occupational physiotherapist, female) |
|  | The delegation of tasks | “We [GPs] are, of course, the spider in the web. Because we deal with many parties [professionals involved]. And I think that this is also our role, or one of our roles.” (GP, male) |
|  | Consult with other professionals in real life or by telephone | “Just the fact that you're speaking to someone on the phone means you at least have a voice to connect with. You can have a more open, informal conversation, yes, by making a phone call. Yes, I say, then you have, you have a voice to go with it. So, I think for situations where online communication isn't really effective, you can also achieve that through a phone call.” (GP, female) |
| 3. Digital care solutions for safe and efficient information exchange | Digital solution to streamline informed consent | “Have you ever considered how nice it would be if, with your insurance card, perhaps with a chip on it, you could be at a doctor's office and simply place your card on a device or nowadays use your phone to grant permission by entering a PIN or something similar? Then you'd say, 'I give permission for you as a doctor to share information with so-and-so,' and then it's all arranged. Essential parts of the medical dossier would be shared because we don't need to know everything, right? […] This would make it easier to consult.” (Occupational physician, male) |
|  | Digital cross-sector platform | “Well, speaking for my line of work, I think that direct emails or some kind of chat function with my colleagues who are treating our […] would be beneficial. It’s rather informal and quick, and we could get very timely, up-to-date information.” (Insurance physician, female) |
|  | Digital directory of contact details | “And what also helps with communication is knowing who is located where, how they can be reached, and when they want to be reached. For our region, we now have a sort of digital directory, which is really helpful when it's filled out, because then you know their working days, when they are available, and when they prefer to be called. All that information can be filled in there.” (GP, female) |
|  | Enable person-centeredness | “Sometimes you just need information, and I find that the way you compose your letter also influences whether others feel you are worth investing energy in. Whether it's a question of willingness, coordination, information, or whatever else—if you put yourself in their shoes, you understand the need to set the context for why you are asking [information]. When you do this, I think you're less likely to be overlooked. I've had some really pleasant conversations with general practitioners, where we genuinely deliberate on what to do and when, discussing who will do what and then planning to revisit the discussion in a month.” (Occupational physician, female) |
|  | Role-specific access rights | “But that is something that must be kept separate. What an occupational physician might know, should not be accessible to an employer. Because that information is not intended for the employer to see.” (GP, female) |
|  | Enable patients’ ownership of their personal health information | “In Belgium, the patient owns his own medical dossier. This means that he has everything, and when a patient goes through an insurance physician, the insurance physician is allowed to see everything. It might seem surprising that patients would be okay with this, but it actually benefits them because then all the information is available to make better decisions. In many cases, decisions have to be made without having all the desired information. The idea is to break down the barriers between different doctors. So, there's one medical dossier, and if the patient allows the doctor to access this dossier, then every doctor knows what they need to know. This system ensures that medical professionals are not working in isolation, improving the continuity and quality of care.” (Insurance physician, male) |
