# Supplementary material for: Exploring motivations, barriers and solutions for interdisciplinary practice in work-focused healthcare: a qualitative study among Dutch healthcare professionals
Source: BMJ Open. 2026 Mar 19;16(3):e103881. doi: 10.1136/bmjopen-2025-103881 (PMC13007094; doi:10.1136/bmjopen-2025-103881)
Supplement: Supplementary file 4 [file bmjopen-2025-103881.reviewer_comments.pdf]

PEER REVIEW HISTORY

BMJ Open publishes all reviews undertaken for accepted manuscripts. Reviewers are asked to complete a checklist review form and are provided with free text boxes to elaborate on their assessment. These free text comments are reproduced below.

ARTICLE DETAILS

Title (Provisional)

Exploring motivations, barriers, and solutions for interdisciplinary practice in work-focused healthcare: A qualitative study among Dutch healthcare professionals

Authors

Zipfel, Nina; Colkesen, B.E.; Hagendijk, Marije E.; Melles, Marijke; van der Burg-Vermeulen, Sylvia J.

VERSION 1 - REVIEW

Reviewer 1

Name Linge, Anita

Affiliation Volda University College Faculty of Social Sciences and History, Faculty of Social Sciences and History

Date 24-May-2025

COI None

Thank you for the opportunity to review this manuscript, which focuses on an important topic of work-focused healthcare. This is an important and timely topic, and I am pleased to see that the authors are investigating this area. Unfortunately, I need to recommend rejecting the article. Abstract, background, and methods need to undergo significant revision. The result section is insufficient and shallowly written and lacks depth and reflection. Working in multidisciplinary teams to better communicate about the patient's goal is not new, and the World Health Organization has promoted this stance since 2010. Additionally, essential references to understand the importance of working in teams are lacking.

Adding some references that may be useful:

Martimo K-P, Takala E-P. Concepts of Work Ability in Rehabilitation. In: Bültmann U, Siegrist J, editors. Handbook of Disability, Work and Health: Springer International Publishing; 2020. p. 551-70.

Organization WH. International Classification of Functioning, Disability and Health: ICF. Geneva: World Health Organization; 2001.

Loisel P, Côté P. The work disability paradigm and its public health implications. In: Patrick L, Johannes A, editors. Handbook of work disability. New York: Springer; 2013. p. 59-67.

Loisel P, Buchbinder R, Hazard R, Keller R, Scheel I, van Tulder M, et al. Prevention of work disability due to musculoskeletal disorders: The challenge of implementing evidence. J Occup Rehabil. 2005;15(4):507-24.

**Reviewer**	**2**
**Name**	**Jensen, Chris**
**Affiliation**	**6. National Centre for Occupational Rehabilitation**
**Date**	**19-Jun-2025**
**COI**	**None**

This is a nice study on the benefits and challenges of the Dutch system on work-focused healthcare. The slutions you suggest are obvious, though somewhat general, and it would require a large effort to implemnt such solutions on a national level. A dgital platform for information exchange would facilitate information transfer, but I have no knowledge of any digital solution that accomplish this because the legislative barriers between health and work domains will always prohibit such information. On the other hand, some improvement on this matter isneeded and possible.

The main weakness of the study is the use of students for interviews and researchers for analyses, but I was convinced that your procedures to deal with this were sound and sufficient.

**Reviewer**	**3**
**Name**	**Guo, Yujie**
**Affiliation**	**Nantong University Medical School**
**Date**	**27-Aug-2025**
**COI**	**None**

Overview and general recommendation:

This study employed qualitative interviews to explore the needs, barriers, and solutions for multidisciplinary collaboration in occupational health care. After careful review of the article, I find the research topic highly innovative, addressing gaps in integrated medical practice within the occupational health field.

The study, set within the context of the Dutch healthcare system, distilled 11 core themes from

qualitative interview data collected from 22 healthcare professionals. It explicitly proposed actionable recommendations such as streamlining communication processes and establishing digital collaboration platforms.The paper maintains rigorous structure, with its methodology section detailing the multi-stage coding process for thematic analysis, thereby enhancing the reliability of findings. Particularly noteworthy is the authors' adoption of patient work engagement as a core metric, aligning with the global shift toward patient-centered occupational health services and offering significant practical guidance.

Although the overall research design is sound, the study exhibits certain shortcomings in its background, methodology, results, and discussion sections, primarily concerning sample representativeness and data saturation.The results section provides a rather general description of specific interventions and strategies, lacking sufficient detail and in-depth elaboration. This makes it difficult for readers to fully grasp the specific content and implementation methods of each measure. In the discussion section, the exploration of the practical impact of the solutions in clinical practice is not sufficiently thorough. A more comprehensive and in-depth analysis of these aspects would further enhance the quality of the article.

Given these factors, I recommend the following revisions: First, supplement the Methods section with the interview outline, data saturation explanation, and other relevant details. Second, conduct more in-depth coding and analysis of the interview content to fully demonstrate the study's value. Addressing these issues will enable the author to further solidify the paper's contribution to the field and its relevance to readers.I will elaborate on my concerns and suggestions in greater detail below. Please address each of my review comments specifically in your response.

Major comments

Title：

1.P1, lines 1-3: The title is too long and should be shortened.

Introduction:

1. P3, lines 72-78: Lack of detailed elaboration on the shift in health concepts: While Huber et al.’s 2009 redefinition of health is mentioned, the specific implications and significance of this shift are not thoroughly explored. This hinders readers’ comprehensive understanding of the importance of the transition from “mere absence of disease” to “capacity for adaptation and self-management.” Supplementary discussion is recommended.

2. P5, Line 86: The background section mentions the benefits of sustainable employability but fails to provide a brief overview of the current situation regarding patients returning to work—such as existing challenges—to justify why multidisciplinary healthcare professionals are needed to provide professional support. Please explain and supplement this.

3. P6, line 113: "Work-focused healthcare" and "occupational healthcare" have similar meanings; please clarify the distinction.

4. The literature review in the research background section lacks depth, and the statement of research value is unclear.

Methods:

1. P7, Lines 136-140: The impact of the unique characteristics of the Dutch occupational health system (e.g., separation of treatment and assessment) on the research questions is not explicitly stated. We recommend adding a description of the specific features of the Dutch occupational health system, particularly how the separation of treatment and assessment influences the study design, sample selection, and interpretation of results. This will help readers better understand the research context and its relevance within their own or international settings.

2. P9, Line 183: Student-led interviews may introduce bias. Clarify how consistency was ensured through training (e.g., simulated interviews) and quality control (e.g., audio review).

3. P8, line 164: The study does not explain why these four groups (general practitioners/occupational physicians/insurance physicians/occupational therapists) were selected (do they cover key decision-makers?). Additionally, why were nursing staff not included? The authors should provide relevant explanations.

4. P9, Line:179: Please clarify whether pre-interviews were conducted prior to formal interviews, the number of interviews conducted, and whether the interview outline was modified based on pre-interview results.

5. P9, Lines 186-189: Interview Guide: Did you provide an incomplete list of questions (e.g., only three examples) or were these the only three questions used? Can you guarantee the results achieved saturation? Please supplement with an appendix and provide an explanation.

6. P10, Line 226: The statement "saturation was not strictly pursued" is mentioned, but the sufficiency of the 22-person sample requires justification. Alternatively, was the interview process terminated because the final three interviewees provided consistent content with earlier interviews? Please provide an explanation.

Results:

1. P12, Line 262: The classification of thematic codes is unreasonable. This section primarily

addresses needs related to communication and collaboration, yet only one theme mentions such needs, resulting in insufficient content. The other two codes are unrelated to this theme. Please provide an explanation.

2. P14, Line 299: We suggest listing “Experienced barriers in current communication and collaboration between professionals” as Theme 1 and “Needs regarding communication and collaboration” as Theme 2. This sequence would first explore existing obstacles, then delve into healthcare professionals' specific needs, and finally propose solutions.

3. P17, Line 374: The second theme mentions “Limiting rules and regulations,” yet the third theme fails to address policy support required from healthcare institutions and other organizations. Does this indicate data saturation? Please provide clarification.

Discussion:

1. The Discussion section provides a preliminary analysis of the current findings, but it is recommended to incorporate more critical thinking, such as exploring inconsistencies with other studies, analyzing potential reasons, and suggesting future research directions.

2. It is suggested to integrate the research findings with clinical practice, discussing how the proposed interventions can be applied in real-world settings and the potential challenges that may arise.

3. The Discussion section lacks coverage of Theme 1; authors are advised to supplement this section.

VERSION 1 - AUTHOR RESPONSE

Reviewer: 1
Mrs. Anita Linge, Volda University College Faculty of Social Sciences and History
Comments to the Author:
Thank you for the opportunity to review this manuscript, which focuses on an important topic of work-focused healthcare. This is an important and timely topic, and I am pleased to see that the authors are investigating this area. Unfortunately, I need to recommend rejecting the article. Abstract, background, and methods need to undergo significant revision. The result section is insufficient and shallowly written and lacks depth and reflection. Working in multidisciplinary teams to better communicate about the patient's goal is not new, and the World Health Organization has promoted this stance since 2010. Additionally, essential references to understand the importance of working in teams are lacking. Adding some references that may be useful:
Martimo K-P, Takala E-P. Concepts of Work Ability in Rehabilitation. In: Bültmann U, Siegrist J, editors. Handbook of Disability, Work and Health: Springer International Publishing; 2020. p. 551-70.
Organization WH. International Classification of Functioning, Disability and Health: ICF. Geneva: World Health Organization; 2001.
Loisel P, Côté P. The work disability paradigm and its public health implications. In: Patrick L, Johannes A, editors. Handbook of work disability. New York: Springer; 2013. p. 59-67.
Loisel P, Buchbinder R, Hazard R, Keller R, Scheel I, van Tulder M, et al. Prevention of work disability

due to musculoskeletal disorders: The challenge of implementing evidence. J Occup Rehabil. 2005;15(4):507-24.

Response: Thank you for your thoughtful review and for highlighting the extensive literature on multidisciplinary teamwork in occupational healthcare. We would like to clarify that our study does not aim to reiterate the importance of multidisciplinary teamwork, which is indeed well-established in both policy and practice.

With our study, we aim to add to this knowledge base by exploring the needs and practical realities of achieving interdisciplinary collaborative practice, focusing on collaboration and communication across healthcare and occupational health. These insights are essential for developing more integrated care pathways that reflect the complexity of work participation challenges. While the "why" of collaborative practice is well-supported in existing literature, our study also contributes to the understanding of the "how", "what" and "when", by identifying specific needs, barriers, enablers, and conditions that professionals encounter in real-world interdisciplinary collaboration. This includes structural, regulatory, communicative, and role-related challenges.

We recognize that the manuscript needs revision to better articulate this focus. In response to your feedback, in the background, we have expanded to better contextualise our study within the broader literature on interdisciplinary collaboration, work disability, and return-to-work. Specifically, we integrated references suggested by the reviewer, including the Work Disability Paradigm and the ICF framework. These additions more clearly position our study within existing conceptual models and public health frameworks. We also added discussion of sustainable employability and work ability as key concepts shaping current approaches to work-focused healthcare.

The following text was added: "This perspective is supported by the World Health Organization's International Classification of Functioning, Disability and Health (ICF), which conceptualises health in terms of functioning and participation, including work participation as a critical domain of life of an individual (9). Furthermore, the concept of work ability has also been proposed as a central outcome and organising principle (10). It emphasises the dynamic balance between a person's health, functional capacities, and the demands of their work, and highlights the role of personal as well as environmental factors in achieving sustainable work participation. Responding effectively to changes in work ability requires coordination between different healthcare professionals, as both medical and contextual factors should be addressed (11). This is further supported by the so-called 'work disability paradigm', which describes work disability not merely as a result of illness or injury, but as a complex interaction of personal, workplace, healthcare, compensation, and societal factors (11). Preventing unnecessary work disability, thus, requires a system-level response that involves multiple stakeholders." And: "Previous research has shown that sustainable work participation outcomes cannot be achieved through intervention from one domain only, it being from the curative care or work-focused healthcare domain (18)."

The references provided also inspired a meaningful addition to the Discussion section: "Beyond the focus on collaboration and communication between professionals in work-focused healthcare, it is important to acknowledge that improving work participation and preventing work disability requires coordinated efforts that extent beyond professionals stakeholders alone (18). Work disability results from a complex interaction of personal,

workplace, healthcare, compensation, and societal factors. Therefore, sustainable solutions should also take into account the engagement of policymakers, employers, the social environment of a person, and other institutional stakeholders responsible for shaping this complex system (11, 18). This makes an integrated care model even more relevant, as they should be embedded within supportive organisational and regulatory environments that can address the complexities of work disability. Previous research has shown that certain medical or non-medical interventions can be effective in tackling work participations outcomes, but their implementation remains challenging (18). Future strategies, should therefore, involve all relevant stakeholders beyond the work-focused healthcare professionals (18)."

Reviewer: 2
Dr. Chris Jensen, 6. National Centre for Occupational Rehabilitation
Comments to the Author:
This is a nice study on the benefits and challenges of the Dutch system on work-focused healthcare.

Response: We thank the reviewer for the positive feedback on the study and its relevance to the Dutch work-focused healthcare system. We appreciate the constructive comments and would like to address the concerns raised.

The solutions you suggest are obvious, though somewhat general, and it would require a large effort to implement such solutions on a national level. A digital platform for information exchange would facilitate information transfer, but I have no knowledge of any digital solution that accomplish this because the legislative barriers between health and work domains will always prohibit such information. On the other hand, some improvement on this matter is needed and possible.

Response: We appreciate the reviewer's critical note regarding the conditional success of implementing a digital platform. In agreement with this observation, we acknowledge that for a digital solution to function effectively in such a complex system legal, technical and institutional barriers must be addressed. To reflect this in the manuscript, we have added the following text in the Implications section of the Discussion on: "However, the feasibility of such a digital platform depends on its alignment with existing legislative frameworks, interoperability with current systems, and acceptance among professionals. These legal, technical and organizational constraints may pose significant barriers that must be considered in the design and implementation of a digital solution."

The main weakness of the study is the use of students for interviews and researchers for analyses, but I was convinced that your procedures to deal with this were sound and sufficient.

Response: To further address the concern raised, we have further expanded the Methodological considerations section to clarify how the students were supervised. Specifically, we emphasise that the full transcripts were reviewed and thematically analysed by the first and second author with extensive experience in qualitative research. The following text was added: "The interviewers also received supervised instructions to promote consistency in interview conduct. Additionally, the full transcripts were reviewed and thematically analysed by the first author and second author, both experienced in qualitative research, providing an additional consistency check to ensure coherence in the analysis across interviews. Nonetheless, subtle differences in tone or follow-up questioning may have influenced the depth or focus of some responses."

Reviewer: 3
Dr. Yujie Guo, Nantong University Medical School, Nantong University School of Medicine
Comments to the Author:
Overview and general recommendation:
This study employed qualitative interviews to explore the needs, barriers, and solutions for multidisciplinary collaboration in occupational health care. After careful review of the article, I find the research topic highly innovative, addressing gaps in integrated medical practice within the occupational health field.

The study, set within the context of the Dutch healthcare system, distilled 11 core themes from qualitative interview data collected from 22 healthcare professionals. It explicitly proposed actionable recommendations such as streamlining communication processes and establishing digital collaboration platforms.The paper maintains rigorous structure, with its methodology section detailing the multi-stage coding process for thematic analysis, thereby enhancing the reliability of findings. Particularly noteworthy is the authors' adoption of patient work engagement as a core metric, aligning with the global shift toward patient-centered occupational health services and offering significant practical guidance.

Although the overall research design is sound, the study exhibits certain shortcomings in its background, methodology, results, and discussion sections, primarily concerning sample representativeness and data saturation.The results section provides a rather general description of specific interventions and strategies, lacking sufficient detail and in-depth elaboration. This makes it difficult for readers to fully grasp the specific content and implementation methods of each measure. In the discussion section, the exploration of the practical impact of the solutions in clinical practice is not sufficiently thorough. A more comprehensive and in-depth analysis of these aspects would further enhance the quality of the article.

> Response: We thank the reviewer for their thoughtful and constructive comments, as well as for recognizing the relevance and innovative contribution of our study to the field of occupational health and integrated care. We especially appreciate the positive assessment of our methodological rigour and the practical relevance of our findings.
>
> We have carefully considered the points raised. Below, we provide a point-by-point response to each concern raised and explain how we have revised the manuscript accordingly.

Major comments
Title：
1. *.P1, lines 1-3: The title is too long and should be shortened.*

> Response: Thank you for this suggestion. We agree that the original title was overly long and have revised it accordingly. The new title is more concise while retaining the key focus of the study: “Exploring needs, barriers, and solutions for interdisciplinary practice in work-focused healthcare: A qualitative study among Dutch healthcare professionals”

Introduction:
1. *P3, lines 72-78: Lack of detailed elaboration on the shift in health concepts: While Huber et al.’s 2009 redefinition of health is mentioned, the specific implications and significance of this shift are not thoroughly explored. This hinders readers’ comprehensive understanding of the importance of*

the transition from “mere absence of disease” to “capacity for adaptation and self-management.” Supplementary discussion is recommended.

Response: We have elaborated on the significance of the health concept redefinition in the revised background section. Specifically, we added: "This redefinition moved the focus of healthcare beyond diagnosis and cure by putting greater emphasis on personal functioning, participation in the labour market, and the dynamic interaction between individuals and their environment. Adaptation, here, refers to a person’s capacity to adjust to physical limitations, emotional stressors, or social barriers, while self-management means the ability to take control of one’s own health, including decision-making, self-monitoring, and interaction with care providers."

We further expanded this argument in the Background section: "This perspective is supported by the World Health Organization’s International Classification of Functioning, Disability and Health (ICF), which conceptualises health in terms of functioning and participation, including work participation as a critical domain of life of an individual. Furthermore, the concept of work ability has also been proposed as a central outcome and organising principle. It emphasises the dynamic balance between a person’s health, functional capacities, and the demands of their work, and highlights the role of personal as well as environmental factors in achieving sustainable work participation. Responding effectively to changes in work ability requires coordination between different healthcare professionals, as both medical and contextual factors should be addressed. This is further supported by the so-called ‘work disability paradigm’, which describes work disability not merely as a result of illness or injury, but as a complex interaction of personal, workplace, healthcare, compensation, and societal factors. Preventing unnecessary work disability, thus, requires a system-level response that involves multiple stakeholders."

We believe this offers a stronger foundation for the relevance of our study.

2. *P5, Line 86: The background section mentions the benefits of sustainable employability but fails to provide a brief overview of the current situation regarding patients returning to work—such as existing challenges—to justify why multidisciplinary healthcare professionals are needed to provide professional support. Please explain and supplement this.*

   Response: We agree that providing more background on the challenges patients face when returning to work strengthens the rationale for multidisciplinary support. While we had already addressed some of these issues, we have now expanded the section to clarify the current situation more explicitly. The following sentence was added: “However, despite the recognised importance of supporting sustainable employability, patients continue to experience difficulties in returning to work. Previous research has shown that patients with chronic illnesses often feel responsible for coordinating their own care, especially when curative and occupational health professionals provide fragmented care without shared protocols and without a responsible person taking lead of the process such as supervisors (16)."

3. *P6, line 113: “Work-focused healthcare” and “occupational healthcare” have similar meanings; please clarify the distinction.*

   Response: We have clarified the distinction in the background section by adding that work-focused healthcare refers to care from both curative and occupational domains that supports

sustainable employability, whereas occupational healthcare is more narrowly focused on employer-commissioned services. The following has been added: "In this context, work-focused healthcare is defined as the advice, treatment, guidance, and support received by patients with work participation problems, from both occupational health professionals and curative care professionals (5)." And: "(5). This broad scope distinguishes it from occupational healthcare, which often is limited to services provided by occupational physicians (5)."

4. *The literature review in the research background section lacks depth, and the statement of research value is unclear.*

   Response: We have revised several parts of the background section including more relevant literature on concepts such as the International Classification of Functioning, Disability and Health (ICF) to further support the importance of improving sustainable employability. We explicitly highlight the empirical gap in understanding professionals' perspective on collaboration in work-focused healthcare. We also clarify how this study contributes to developing more integrated care strategies to support sustainable work participation. We added the following: "By exploring the needs, barriers, and potential solutions, this study contributes to the development of more integrated strategies to support better work participation and more effective interdisciplinary practice."

Methods:

1. *P7, Lines 136-140: The impact of the unique characteristics of the Dutch occupational health system (e.g., separation of treatment and assessment) on the research questions is not explicitly stated. We recommend adding a description of the specific features of the Dutch occupational health system, particularly how the separation of treatment and assessment influences the study design, sample selection, and interpretation of results. This will help readers better understand the research context and its relevance within their own or international settings.*

   Response: The comment has triggered us to add background information, specifically on the role of GPs and physiotherapists in the Background section: "(5). Especially, the role of the general practitioners alongside occupational health professionals has been recognized to contribute to better work participation as they act as the first point of contact for navigating between different sectors (15, 16). Moreover, professionals such has physiotherapists have been found to be of added-value in providing work-focused healthcare (17). Yet, those professioals are often not optimally integrated into work-focused healthcare pathways." We also added this to the Method section: "The division between curative and work-focused healthcare shaped participant selection, as it highlights the need to capture perspectives from both domains. Therefore, general practitioners were intentionally included from curative care due to their unique position as the first point of contact in curative care. In the Dutch healthcare system, GPs often remain involved throughout a patient's care trajectory. Although GPs do not formally lead the RTW process, their role in early identification and patient relationship is essential. Occupational physiotherapists were chosen due the growing recognition of the importance of physiotherapists in the work-focused healthcare trajectories."

2. *P9, Line 183: Student-led interviews may introduce bias. Clarify how consistency was ensured through training (e.g., simulated interviews) and quality control (e.g., audio review).*

   Response: We indeed took measures to ensure consistency such as simulated interviews after which the interview guides were harmonized and a random revision of the audio

recordings. We added this information in the Methods: “To ensure alignment and standardisation, students presented preliminary versions of their interview guides, which were reviewed with constructive feedback from the first and fourth author (NZ and MM). Additionally, the students conducted simulated interviews prior to the formal data collection to support consistency in interview technique.” And: “The research team was able to randomly review a subset of audio recordings to check for consistency in delivery and adherence to the interview guide.” Yet, we do recognize this approach as a possible threat to bias, which is why we also further elaborate on this in the Discussion section: “The interviewers also received supervised instructions to promote consistency in interview conduct. Additionally, the full transcripts were reviewed and thematically analysed by the first author and second author, both experienced in qualitative research, providing an additional consistency check to ensure coherence in the analysis across interviews. Nonetheless, subtle differences in tone or follow-up questioning may have influenced the depth or focus of some responses.”

3. *P8, line 164: The study does not explain why these four groups (general practitioners/occupational physicians/insurance physicians/occupational therapists) were selected (do they cover key decision-makers?). Additionally, why were nursing staff not included? The authors should provide relevant explanations.*

   Response: Please see our response under point 1. We also added the following in the Discussion section: “In addition, although this study included key professionals from both curative and work-focused healthcare, it may not fully capture the perspectives of all relevant stakeholders involved in work-focused healthcare, such as psychologists, medical specialists, nursing staff, or employers. This selection of the four groups was based on their central role in decision-making and coordination within the Dutch system, as well as feasibility considerations. Nevertheless, future research may consider including a broader range of professionals to further enrich the understanding of interdisciplinary collaboration in work-focused healthcare.”

4. *P9, Line:179: Please clarify whether pre-interviews were conducted prior to formal interviews, the number of interviews conducted, and whether the interview outline was modified based on pre-interview results.*

   Response: The students conducted per group one simulated interview prior to formal Interviews. The interview guide was changed after feedback by the first and fourth author to ensure alignment. Please see our reaction under point 2.

5. *P9, Lines 186-189: Interview Guide: Did you provide an incomplete list of questions (e.g., only three examples) or were these the only three questions used? Can you guarantee the results achieved saturation? Please supplement with an appendix and provide an explanation.*

   Response: Please find the interview guide in supplementary file 2. Moreover, the goal was not to reach data saturation, but to obtain a broad and representative overview of professional perspectives across the relevant disciplines. We reflected on this potential limitation in the Discussion section: “A further limitation concerns the sample size and thematic saturation. While the exploratory approach allowed for a broad and inclusive set of perspectives across multiple professional groups, it may have come at the expense of deeper exploration within each group. As a result, certain aspects specific to particular professional roles may have remained underexplored. Although few new insights emerged in the final interviews per group, the absence of a formal saturation assessment may limit the extent of thematic completeness.

Future research could adopt an approach driven by saturation or include a larger sample size per professional group to ensure both breadth and depth of perspectives."

6. *P10, Line 226: The statement "saturation was not strictly pursued" is mentioned, but the sufficiency of the 22-person sample requires justification. Alternatively, was the interview process terminated because the final three interviewees provided consistent content with earlier interviews? Please provide an explanation*.

   Response: We clarified in the Methods section that our goal was to include five professionals per group, which we considered sufficient to ensure diversity in perspectives across the involved disciplines. While one group included additional participants, the others did not due to time and feasibility constraints. A formal saturation assessment was not conducted, but the number of interviews allowed us to explore the key issues relevant to our research question. The following was added: "The goal was to include five professionals per group as this would offer sufficient diversity to identify key themes. While one student group included additional participants, the other groups did not expand further due to time and feasibility constraints."

Results:

1. *P12, Line 262: The classification of thematic codes is unreasonable. This section primarily addresses needs related to communication and collaboration, yet only one theme mentions such needs, resulting in insufficient content. The other two codes are unrelated to this theme. Please provide an explanation.*

   Response: We appreciate your thoughtful feedback regarding the thematic classification in this section. We appreciate your concern and would like to clarify our approach.
   The theme 'Needs regarding communication and collaboration' was derived from a consistent and recurring pattern in the data, where professionals expressed various needs related to improving interdisciplinary interaction. To structure these findings meaningfully, we identified three interrelated codes that represent distinct dimensions of this overarching need. However, we acknowledge that the current labeling may have caused confusion. To improve clarity and better reflect their connection to the theme, we have revised the code labels as follows: 1. Motivations for interdisciplinary communication and collaboration; 2. Conditions for effective communication and collaboration; and 3. Timing and triggers for interdisciplinary communication and collaboration.

2. *P14, Line 299: We suggest listing "Experienced barriers in current communication and collaboration between professionals" as Theme 1 and "Needs regarding communication and collaboration" as Theme 2. This sequence would first explore existing obstacles, then delve into healthcare professionals' specific needs, and finally propose solutions.*

   Response: We appreciate the reviewer's suggestion to reorder the themes. We considered this structure during our analysis, However, we chose to present the needs first, as this approach offers contextual grounding and helps to establish whether professionals perceive a need for improved collaboration in the first place. Starting with the needs allows us to frame the analysis more positively and explore why communication and collaboration matter, what professionals hope to achieve in terms of ideal collaboration, before introducing the barriers that prevent these needs from being met. We believe this sequencing provides a constructive flow from aspiration (needs), to challenge (barriers), to improvement (solutions). To make this more explicit we added the following in the results: "This structure was chosen in order to first

illustrate the perceived relevance and urgency of the topic before the specific barriers and solutions."

3. *P17, Line 374: The second theme mentions "Limiting rules and regulations," yet the third theme fails to address policy support required from healthcare institutions and other organizations. Does this indicate data saturation? Please provide clarification*

   Response: It is correct that Theme 3, which focuses on solutions, does not explicitly address structural or policy-level responses to the regulatory constraints identified in Theme 2. This reflects the nature of our interview data: participating professionals primarily proposed solutions that were actionable within their own influence. Policy-level changes were not directly raised, likely because such changes are perceived to fall outside their professional scope or mandate. However, we agree that addressing regulatory and institutional barriers is essential for systemic improvement. Therefore, we have added a paragraph to the Discussion section acknowledging this gap and emphasising the importance of broader stakeholder involvement to complement the professional-level solutions reported in our study: "While our study highlighted a range of practical and professional-level solutions, it is important to note that regulatory constraints would also need to be addressed to achieve systemic improvement. The absence of system-level solutions in our study may reflect a perceived lack of mandate or influence over policy among professionals. However, previous research emphasised that work disability is shaped by a complex interplay between personal, clinical, workplace, and systemic factors, including legislative structures, requiring integrated solutions beyond the professional level (13, 24). The limited attention to system-level solutions in our findings highlights the importance of involving policymakers and other stakeholders in future co-creation processes to support sustainable implementation of interdisciplinary practice within integrated work-focused healthcare."

Discussion:

1. *The Discussion section provides a preliminary analysis of the current findings, but it is recommended to incorporate more critical thinking, such as exploring inconsistencies with other studies, analyzing potential reasons, and suggesting future research directions.*

   Response: Thank you for your suggestions to strengthen the Discussion section. By carefully considering and addressing the various previous reviewer comments throughout the revision process, we believe we have significantly enhanced the analytical depth, critical reflection, and relevance of the Discussion section.

2. *It is suggested to integrate the research findings with clinical practice, discussing how the proposed interventions can be applied in real-world settings and the potential challenges that may arise.*

   Response: We agree that discussing practical implementation is an important aspect of the study's relevance. We, therefore, added a short paragraph to the Discussion section that addresses how the proposed solutions can be embedded in daily practice: "To translate the findings of our study into practice, proposed solutions such as digital platforms, central coordination roles, and interprofessional guidelines, should be embedded within existing workflows and systems. This requires not only technological infrastructure and interoperability, but also alignment with legal and regulatory frameworks, compliance with privacy and data protection standards, and ensuring professional role clarity to avoid overlap or ambiguity in responsibilities Future research on implementation strategies, including facilitation,

involvement of end-users, training and feedback mechanisms, can support successful and sustainable uptake in the future. Moreover, co-creation with stakeholders at multiple levels, including policymakers and institutional leaders, will be essential to ensure that solutions are not only feasible but also scalable and aligned with broader system goals (49, 50)."

We believe that by incorporating this and other reviewer feedback, the revised Discussion section now offers a more comprehensive and practice-oriented perspective that better reflects real-world applicability.

3. *The Discussion section lacks coverage of Theme 1; authors are advised to supplement this section.*

   Response: Thank you for the helpful suggestion. We agree that Theme 1 (needs regarding communication and collaboration) was not sufficiently reflected in the Discussion section. We therefore added a paragraph early in the Discussion section summarising professionals' motivations for interdisciplinary collaboration, the identified conditions for effective communication, and the importance of early and preventive coordination in the care trajectory. The paragraph we added: "Professionals in this study, emphasised that effective work-focused healthcare depends on early, coordinated, and person-centred communication between professionals. The need for better interdisciplinary collaboration was driven by the shared goal of improving patients' long-term work participation, particularly for those with chronic or complex conditions where fragmented communication can delay recovery or lead to inconsistent support. Similar to previous research, professionals considered timely and proactive contact, especially early on in the patient's trajectory, as essential to prevent stagnation and support continuity of care (5, 17, 18). Collaboration should be guided by clear coordination structures and mutual understanding of roles to ensure coherent decision-making (18, 30, 31)."

VERSION 2 - REVIEW

Reviewer **1**

Name **Linge, Anita**

Affiliation **Volda University College Faculty of Social Sciences and History, Faculty of Social Sciences and History**

Date **25-Nov-2025**

COI

Thank you for the opportunity to review this article again. The authors have made several changes in the manuscript which has undergone important changes. However, I still have significant concerns regarding the results section. In my previous review, I recommended rejecting the article due to major issues, and I note that Reviewer 3 supported some of these concerns. After carefully re-reading the revised manuscript, I continue to have serious reservations about the results section.

Abstract:

Page 2, line 45: I suggest making the sentence clearer by exclude “the n=22 interviews” since this information stands in the section above.

Page 12, lines 286. This section should be moved to line 265 to give a more holistic image of the participants.

Results

This section raises significant concerns due to its overly general descriptions, insufficient detail, and lack of in-depth elaboration. I continue to find the content difficult to fully comprehend, particularly with respect to the implications of the reported results. In my previous review, I noted major issues, many of which were also highlighted by Reviewer 3. Unfortunately, most of these concerns remain unresolved in this draft.

The themes are presented only briefly, without sufficient analytical depth or interpretive insight. The analysis does not adequately explore how professionals articulate their understanding of needs, barriers, and solutions.

Regarding the use of quotes, they are presented without substantive analytical reflection. Many quotations are too brief or fail to clearly support the thematic categories. For example, the statement *“if we abandon this effective system…”* appears to concern withdrawal from an ineffective system rather than motivation for interdisciplinary communication and collaboration.

Results: “Needs regarding communication and collaboration”

- Subtheme 1 (“Motivation for interdisciplinary communication and collaboration”)—apart from the quotation—could serve more appropriately as an introductory framing in the results section.
- Subthemes 2 and 3 appear to represent barriers rather than needs. I recommend relocating them to the “Barriers” section. Both lack sufficient analytical elaboration.

Results: “Experienced barriers in current communication”

- **Subtheme 1:** Although this theme demonstrates a somewhat deeper analytic approach than the previous ones, the quotation suggests that communication does occur, albeit verbally. The text does not sufficiently analyse why this form of communication constitutes a barrier.
- **Subtheme 2:** This theme conflates issues of workload, time constraints, and systemic weaknesses. A more rigorous analysis is needed to clarify how each factor specifically influences communication and collaboration.
- **Subtheme 3:** Greater detail is required regarding how informed consent is practiced. In many cases, verbal consent during consultation may suffice for collaboration. The current analysis lacks reflection on why professionals cannot obtain consent directly from patients. This omission suggests a passive professional stance, which may itself represent a barrier to communication.

- **Subtheme 4:** The theme should specify which communication tools are absent. For instance, if electronic journal systems fail to integrate, this should be clearly distinguished from other ways of communicating.

Results: "Solutions to reach optimal communication"

- **Subtheme 1:** This theme relies on two lengthy quotations but lacks deeper analytical reflection. The text should explain how insight into each other's roles concretely contributes to improved communication and collaboration.
- **Subthemes 2 and 4:** These themes overlap considerably and should be merged. Both currently lack sufficient analytical depth.
- **Subtheme 3:** The title should be revised to "Clearer coordination of care." The theme also requires more rigorous analytical reflection.

VERSION 2 - AUTHOR RESPONSE

Response to Reviewer 1:

Thank you for the opportunity to review this article again. The authors have made several changes in the manuscript which has undergone important changes. However, I still have significant concerns regarding the results section. In my previous review, I recommended rejecting the article due to major issues, and I note that Reviewer 3 supported some of these concerns. After carefully re-reading the revised manuscript, I continue to have serious reservations about the results section.

Response: We sincerely thank the reviewer for taking the time to carefully re-read the revised manuscript and for acknowledging the changes we made. We understand and appreciate the remaining concerns regarding the Results section. Based on this feedback, we have once again substantially revised the section to further strengthen the analytical depth, clarity, and coherence of our findings. We hope the changes now adequately address the concerns raised.

Abstract:

Page 2, line 45: I suggest making the sentence clearer by exclude "the n=22 interviews" since this information stands in the section above.

Response: Thank you for this helpful suggestion. We agree that repeating the "22 interviews" is redundant in this sentence/ We have now revised the sentence accordingly: "Interviews with professionals revealed key needs related to why, what, and when of collaboration and communication in the context of work-focused healthcare."

Page 12, lines 286. This section should be moved to line 265 to give a more holistic image of the participants.

Response: Thank you for this comment. We moved the sentence to the suggested place in the manuscript.

Results

This section raises significant concerns due to its overly general descriptions, insufficient detail, and lack of in-depth elaboration. I continue to find the content difficult to fully comprehend, particularly with respect to the implications of the reported results. In my previous review, I noted major issues, many of which were also highlighted by Reviewer 3. Unfortunately, most of these concerns remain unresolved in this draft.

The themes are presented only briefly, without sufficient analytical depth or interpretive insight. The analysis does not adequately explore how professionals articulate their understanding of needs, barriers, and solutions.

Regarding the use of quotes, they are presented without substantive analytical reflection. Many quotations are too brief or fail to clearly support the thematic categories. For example, the statement *"if we abandon this effective system…"* appears to concern withdrawal from an ineffective system rather than motivation for interdisciplinary communication and collaboration.

Response: We thank the reviewer for this observation. In response, we have substantially revised the Results section to improve analytical depth and clarity. We expanded the thematic narratives, added more interpretive commentary to link quotes to key insights, and clarified the implications of the findings for interdisciplinary communication. These changes aim to provide a more rigorous and coherent presentation of the results.

Results: "Needs regarding communication and collaboration"

- Subtheme 1 ("Motivation for interdisciplinary communication and collaboration")—apart from the quotation—could serve more appropriately as an introductory framing in the results section.

  Response: We thank the reviewer for this suggestion. We have revised the subtheme to improve its analytical depth and to clarify how professionals conceptualise their motivations for interdisciplinary communication. In the revised version, we now present this subtheme as a coherent analytical narrative that explains why professionals across disciplines consider collaboration necessary, particularly in complex or stagnating cases.

We have also expanded the interpretation of the included quote to illustrate the professionals' broader conceptualisation of participation-oriented care, and explicitly contrasted this with institution-centred care. This revised paragraph now also serves as a contextualising introduction to the rest of the results section, in line with the reviewer's advice.

- Subthemes 2 and 3 appear to represent barriers rather than needs. I recommend relocating them to the "Barriers" section. Both lack sufficient analytical elaboration.

Response: We thank the reviewer for this insightful comment. We agree that, as originally formulated, subthemes 2 and 3 are more appropriately conceptualized as barriers rather than needs. While needs and barriers can be understood as two sides of the same coin, where barriers reflect unmet or obstructed needs, we recognize that the analytical emphasis in these subthemes on obstacles rather than on articulated needs is more suited. Accordingly, we have relocated subtheme 2 and 3 to the barriers section and reformulated the needs to clear barriers. The original subtheme "Conditions for effective communication and collaboration" is now presented as "Lack of preconditions for effective communication and collaboration", reframed as a structural and organisational barrier and rewritten to include stronger analytical interpretation. The original subtheme "Timing and triggers for communication and collaboration" is now presented as "Lack of timely and proactive triggers for interdisciplinary communication and collaboration", highlighting the systemic absence of early engagement mechanisms and the resulting reactive nature of collaboration. In both cases, we retained key illustrative quotes and incorporated additional interpretive depth to clarify how these issues hinder effective communication and collaboration in practice.

Results: "Experienced barriers in current communication"

- **Subtheme 1:** Although this theme demonstrates a somewhat deeper analytic approach than the previous ones, the quotation suggests that communication does occur, albeit verbally. The text does not sufficiently analyse why this form of communication constitutes a barrier.

Response: We thank the reviewer for this insightful comment. In the revised manuscript, we have substantially rewritten this subtheme to clarify that verbal communication across domains is not routinely practiced but is instead often perceived as not permitted under current regulatory and consent requirements. We added explanation that uncertainty surrounding the need for targeted patient consent, frequently interpreted as requiring written documentation, leads professionals to refrain from both written and

verbal communications in the absence of formally verifiable consent. As a result, communication is often delayed or avoided altogether rather than substituted by verbal exchange. . Also, we have added interpretive insight into the absence of formalised systems to support written information exchange. We also clarified how diverging treatment goals and the perception of occupational physicians' lack of independence hinder information sharing and collaboration. These additions offer a deeper understanding of the underlying mechanisms that contribute to the barrier of limited shared knowledge and goals.

- **Subtheme 2:** This theme conflates issues of workload, time constraints, and systemic weaknesses. A more rigorous analysis is needed to clarify how each factor specifically influences communication and collaboration.

Response: We have revised this subtheme to disentangle workload, time constraints, and systemic procedural barriers and to provide a more analytical interpretation of how each factor specifically hampers interdisciplinary communication and collaboration. In the revised Results section, we now distinguish between (1) administrative delays caused by formal authorisation procedures, (2) high volumes of information requests and associated documentation burden, and (3) the absence of protected time and structural opportunities for informal peer consultation. We also added interpretive analysis to show how these factors jointly contribute to collaboration fatigue and delayed decision-making, thereby reducing the feasibility and perceived value of interdisciplinary collaboration.

- **Subtheme 3:** Greater detail is required regarding how informed consent is practiced. In many cases, verbal consent during consultation may suffice for collaboration. The current analysis lacks reflection on why professionals cannot obtain consent directly from patients. This omission suggests a passive professional stance, which may itself represent a barrier to communication.

Response: We have revised the section on 'Limiting rules and regulations' to provide a more in-depth analysis of how informed consent procedures influence communication practices. In the revised text, we now explicitly reflect on the cautious or passive stance professionals may adopt in response to legal uncertainty and procedural complexity, even when verbal consent might suffice. We also elaborate on how the lack of integrated systems and structural support exacerbates these challenges.

- **Subtheme 4:** The theme should specify which communication tools are absent. For instance, if electronic journal systems fail to integrate, this should be clearly distinguished from other ways of communicating.

Response: Thank you for your helpful suggestion. We have revised the subtheme now titled 'Insufficient facilities to communicate' to more clearly specify which communication tools are lacking. We elaborated on the absence of integrated electronic health records, the inability to access contact details, and the limitations of secure messaging systems. We also added analytical depth by linking these infrastructural gaps to professional isolation and reduced care coordination.

Results: "Solutions to reach optimal communication"

- **Subtheme 1:** This theme relies on two lengthy quotations but lacks deeper analytical reflection. The text should explain how insight into each other's roles concretely contributes to improved communication and collaboration.

Response: These strategies highlight that improved role clarity does not merely enhance technical coordination but also reduces professional hierarchy and increases perceived legitimacy, both essential to meaningful communication and integrated care.

- **Subthemes 2 and 4:** These themes overlap considerably and should be merged. Both currently lack sufficient analytical depth.

Response: Thank you for this helpful observation. We have merged the two themes ('Facilitate information sharing' and 'Digital care solutions') into a single theme titled 'Digital solutions to facilitate secure and efficient information sharing', to avoid redundancy and improve coherence. In the revised version, we have also added analytical depth by elaborating on how these solutions address legal, relational, and structural barriers, and by interpreting the proposed digital tools as enablers of trust, efficiency, and person-centred collaboration.

- **Subtheme 3:** The title should be revised to "Clearer coordination of care." The theme also requires more rigorous analytical reflection.

Response: Thank you for this helpful suggestion. We have revised the theme title to 'Clearer coordination of care' and expanded the section to include deeper analytical reflection.

Specifically, we now elaborate on how a central care coordinator, task delegation, and real-time consultation were proposed to address inefficiencies in collaboration. These additions clarify how professionals envision improving structural and relational aspects of interdisciplinary communication.

VERSION 3 - REVIEW

**Reviewer**	**1**
**Name**	**Linge, Anita**
**Affiliation**	**Volda University College Faculty of Social Sciences and History, Faculty of Social Sciences and History**
**Date**	**22-Feb-2026**
**COI**	

Thank you for the opportunity to review this manuscript again, which has now undergone a substantial revision. The article addresses an important and timely topic, and I am pleased to see that the authors have made significant improvements, particularly in the results and discussion sections. The revisions have strengthened the manuscript considerably.

At this stage, I have only minor comments. My suggestions below are intended to further clarify the results section and enhance the overall readability and coherence of the manuscript. I hope the authors find them helpful as they prepare the final version.

I have reviewed the revised manuscript with tracked changes, and all line numbers in the comments below refer to that version.

Results Section

- Line 318:

It appears that the analysis consists of three main themes and nine sub-themes. I recommend revising the text to reflect this more accurately by changing “eleven themes” to “three main themes and nine sub-themes.”

- Line 403:

Instead of using the general term knowledge, consider specifying work-related knowledge to increase precision.

- Line 429:

The sentence beginning with “In addition...” should be moved down to form a new paragraph.

- Line 436:

Similarly, please break the text here and begin a new paragraph with the sentence starting with “Finally, several...”.

- Line 483:

The sentence starting with “According to current...” should be moved to a new paragraph to improve readability.

- Line 499:

The sentence beginning with “Professionals experienced...” should also start a new paragraph.

- Line 519:

I suggest deleting this sentence, as the content is repeated and described more clearly in the lines that follow.

- Line 525:

Consider moving this sentence down to start a new paragraph. Additionally, I recommend rephrasing it to begin with “The participants also...”.

VERSION 3 - AUTHOR RESPONSE

Response to Reviewer 1:

Thank you for the opportunity to review this manuscript again, which has now undergone a substantial revision. The article addresses an important and timely topic, and I am pleased to see that the authors have made significant improvements, particularly in the results and discussion sections. The revisions have strengthened the manuscript considerably.

At this stage, I have only minor comments. My suggestions below are intended to further clarify the results section and enhance the overall readability and coherence of the manuscript. I hope the authors find them helpful as they prepare the final version.

I have reviewed the revised manuscript with tracked changes, and all line numbers in the comments below refer to that version.

Response: Thank you for your positive and encouraging feedback on the revised manuscript. We appreciate your careful review and are grateful for your constructive suggestions to further improve the clarity and readability of the results section. Below, we address each of your minor comments in detail and indicate the corresponding revisions made in the manuscript.

Results Section

- Line 318:

It appears that the analysis consists of three main themes and nine sub-themes. I recommend revising the text to reflect this more accurately by changing “eleven themes” to “three main themes and nine sub-themes.”

Response: Thank you for this helpful suggestion. We have revised the text to more accurately reflect the analytical structure and now refer to three main themes and nine subthemes instead of eleven themes.

- Line 403:

Instead of using the general term knowledge, consider specifying work-related knowledge to increase precision.

Response: Thank you for this helpful suggestion. We have revised the wording to “work-related knowledge” to improve precision and better reflect the specific context of collaboration in work-focused healthcare.

- Line 429:

The sentence beginning with “In addition…” should be moved down to form a new paragraph.

Response: Thank you for this suggestion. We have moved the sentence beginning with “In addition…” to a new paragraph to improve the readability and structure of the section.

- Line 436:

Similarly, please break the text here and begin a new paragraph with the sentence starting with “Finally, several…”.

Response: We have revised the text accordingly and now begin a new paragraph with the sentence starting with “Finally, several…” to improve readability and structure.

- Line 483:

The sentence starting with "According to current…" should be moved to a new paragraph to improve readability.

Response: Thank you. We have moved this sentence to a new paragraph to improve readability.

- Line 499:

The sentence beginning with "Professionals experienced…" should also start a new paragraph.

Response: Thank you. We have revised the paragraph structure accordingly.

- Line 519:

I suggest deleting this sentence, as the content is repeated and described more clearly in the lines that follow.

Response: Thank you for this suggestion. We have removed the redundant sentence and revised the paragraph for clarity.

- Line 525:

Consider moving this sentence down to start a new paragraph. Additionally, I recommend rephrasing it to begin with "The participants also…".

Response: Thank you for this suggestion. We have moved the sentence to a new paragraph and revised it to begin with "The participants also…" to improve clarity and readability.
